# Tumor derived exosomal ENTPD2 impair CD8^+^ T cell function in colon cancer through ATP-adenosine metabolism reprogramming

**DOI:** 10.1186/s12964-024-01654-2

**Published:** 2024-05-16

**Authors:** Mengchen Shi, Linsen Ye, Lu Zhao, Lingyuan He, Junxiong Chen, Jingdan Zhang, Yixi Su, Haiyan Dong, Jiaqi Liu, Liumei Liang, Wenwen Zheng, Yanhong Xiao, Huanliang Liu, Xiangling Yang, Zihuan Yang

**Affiliations:** 1 Department of Clinical Laboratory, The Sixth Affiliated Hospital, Sun Yat-sen University, Guangzhou, 510655 China; 2 Guangdong Provincial Key Laboratory of Colorectal and Pelvic Floor Diseases, The Sixth Affiliated Hospital, Sun Yat-sen University, Guangzhou, 510655 China; 3 Biomedical Innovation Center, The Sixth Affiliated Hospital, Sun Yat-sen University, Guangzhou, 510655 China; 4 Department of Hepatic Surgery, The Third Affiliated Hospital, Sun Yat-sen University, Guangzhou, 510630 China

**Keywords:** Colon cancer, Exosome, Adenosine, Ectonucleoside triphosphate diphosphohydrolase, CD8^+^ T cell

## Abstract

**Background:**

Extracellular ATP–AMP–adenosine metabolism plays a pivotal role in modulating tumor immune responses. Previous studies have shown that the conversion of ATP to AMP is primarily catalysed by Ectonucleoside triphosphate diphosphohydrolase 1 (ENTPD1/CD39), a widely studied ATPase, which is expressed in tumor-associated immune cells. However, the function of ATPases derived from tumor cells themselves remains poorly understood. The purpose of this study was to investigate the role of colon cancer cell–derived ATPases in the development and progression of colon cancer.

**Methods:**

Bioinformatic and tissue microarray analyses were performed to investigate the expression of ATPase family members in colon cancer. An ATP hydrolysis assay, high-performance liquid chromatography (HPLC), and CCK8 and colony formation assays were used to determine the effects of ENTPD2 on the biological functions of colon cancer cells. Flow cytometric and RNA-seq analyses were used to explore the function of CD8^+^ T cells. Immunoelectron microscopy and western blotting were used to evaluate the expression of ENTPD2 in exosomes. Double-labelling immunofluorescence and western blotting were used to examine the expression of ENTPD2 in serum exosomes and colon cancer tissues.

**Results:**

We found that ENTPD2, rather than the well-known ATPase CD39, is highly expressed in cancer cells and is significantly positively associated with poor patient prognosis in patients with colon cancer. The overexpression of ENTPD2 in cancer cells augmented tumor progression in immunocompetent mice by inhibiting the function of CD8^+^ T cells. Moreover, ENTPD2 is localized primarily within exosomes. On the one hand, exosomal ENTPD2 reduces extracellular ATP levels, thereby inhibiting P2X7R-mediated NFATc1 nuclear transcription; on the other hand, it facilitates the increased conversion of ATP to adenosine, hence promoting adenosine-A2AR pathway activity. In patients with colon cancer, the serum level of exosomal ENTPD2 is positively associated with advanced TNM stage and high tumor invasion depth. Moreover, the level of ENTPD2 in the serum exosomes of colon cancer patients is positively correlated with the ENTPD2 expression level in paired colon cancer tissues, and the ENTPD2 level in both serum exosomes and tissues is significantly negatively correlated with the ENTPD2 expression level in tumor-infiltrating CD8^+^ T cells.

**Conclusion:**

Our study suggests that exosomal ENTPD2, originated from colon cancer cells, contributes to the immunosuppressive microenvironment by promoting ATP–adenosine metabolism. These findings highlight the importance of exosome-derived hydrolytic enzymes as independent entities in shaping the tumor immune microenvironment.

**Supplementary Information:**

The online version contains supplementary material available at 10.1186/s12964-024-01654-2.

## Introduction

Immune checkpoint-blocking antibody treatment is an effective contributor to a successful therapeutic response, but only a limited patient population exhibits a response to immune checkpoint-blocking antibody treatment for colon cancer [1]. To achieve a robust immunotherapeutic response, it is important to consider the dynamic interactions between tumor cells and immunoregulators in the tumor microenvironment (TME) [2, 3]. Therefore, additional immune checkpoint targets that can elicit efficacious immune responses in the TME are urgently needed.

Adenosine-mediated immunosuppression in the TME has been studied extensively. Targeting adenosine formation and its subsequent signalling events has great potential utility in cancer immunotherapy [4]. Adenosine can be formed via ATP metabolism through a two-step process: an ATPase converts extracellular ATP to 5’-AMP, and an AMPase degrades 5’-AMP to generate adenosine [5]. The expression of ectonucleoside triphosphate diphosphohydrolase 1 (ENTPD1/CD39), which is a well-studied ATPase involved in ATP–adenosine metabolism, has been thoroughly described in tumor-infiltrating immune cells [5, 6]. Less is known regarding the expression of ATPases, including CD39, in colon cancer cells. Bastid [6] demonstrated that the expression of CD39 is low in human colon cancer cells. CD39 belongs to the ENTPD family, which comprises eight members, four (CD39, ENTPD2, ENTPD3 and ENTPD8) of which contain an ATP hydrolytic domain that faces the extracellular space [7]. However, there are no reports on the expression of the remaining ATPases and their immunomodulatory functions within colon cancer cells.

Accumulating evidence shows that tumor-associated proteins anchored on the tumor cell membrane can be released into the TME, where they can induce a series of downstream cascades involving the release of exosomes that alter the function of immunocytes [8]. Exosomes, ranging in size from 30 to 150 nm, serve as carriers of cargos that represents the contents of the origin cells. They have the ability to reshape the TME and accelerate tumor progression [9]. Therefore, the complexity of the interactions among exosomal immune checkpoint proteins, immune cells, and the TME should be taken into consideration when formulating immunotherapeutic strategies.

In this study, for the first time, we performed a screen and demonstrated that ENTPD2 but not other ATPases (CD39, ENTPD3 and ENTPD8) is highly expressed in human colon cancer cells and is closely associated with poor patient prognosis. ENTPD2 promotes tumor progression in vivo by inhibiting the function of CD8^+^ T cells. Furthermore, we found that ENTPD2 is released primarily in the membrane form into the extracellular space of colon cancer cells via exosomes. Exosome-derived hydrolytic enzymes have rarely been studied, and we demonstrated that exosomal ENTPD2 suppressed CD8^+^ T-cell function by both limiting ATP-P2X7 receptor (P2 X7R)-mediated NFATc1 nuclear transcription and promoting adenosine-A2AR pathway activity. In patients with colon cancer, the serum level of exosomal ENTPD2 is increased and positively correlated with advanced TNM stage and high tumor invasion depth. Furthermore, ENTPD2 expression in both serum exosomes and tissues exhibited a significant negative correlation with the abundance of tumor-infiltrating CD8^+^ T cells. Our research demonstrated for the first time that exosomes derived from colon cancer cells carry ENTPD2, which promotes ATP–adenosine metabolism and induces the formation of an immunosuppressive microenvironment.

## Materials and methods

### Human samples and cell lines

We purchased human colon cancer microarray tissue slides from Shanghai Outdo Biotech Inc. (Shanghai, China, Cat# HColA180Su21) for ENTPD2 expression evaluation using immunohistochemical analysis. We also collected peripheral blood samples and tissue sections from colon cancer patients who sought treatment at the Sixth Affiliated Hospital of Sun Yat-sen University, and we received ethical approval from the same university for our research (2022ZSLYEC-24, 2020ZSLYEC-236). Human colon cancer cell lines (RKO and DLD1), as well as the murine colon cancer cell line MC38, were acquired from the American Type Culture Collection (ATCC) and immediately tested for mycoplasma contamination prior to use.

### Cell transfection

To generate ENTPD2-knockdown colon cancer cells, we used lentiviral shRNA transduction. shRNA oligonucleotides targeting human ENTPD2 and vector control shRNA oligonucleotides were inserted separately into the lentiviral vector GV248 (GeneChem, Shanghai, China). The murine Entpd2 sequence was cloned and ligated into the lentiviral vector LV217 (GeneCopoeia, Guangzhou, China). Subsequently, the resulting plasmids were cotransfected with packaging plasmids into 293T cells using Lipofectamine 2000 (Invitrogen, Carlsbad, USA). After 48 h of incubation, colon cancer cells were infected with the packaged lentiviruses prior to selection with puromycin for 2 weeks. siRNAs targeting ENTPD2 (siENTPD2) and ENTPD8 (siENTPD8) were designed and purchased from RiboBio (China). Colon cancer cells were transfected with the siRNAs using Lipofectamine® RNAiMAX Reagent (Invitrogen, Carlsbad, USA). The efficacies of ENTPD2 knockdown and overexpression were assessed via quantitative real-time polymerase chain reaction (qRT–PCR) and western blotting.

### Quantitative real-time polymerase chain reaction

Total RNA was extracted from colon cancer cells by TRIzol (Invitrogen, Carlsbad, USA), and cDNA was prepared using a cDNA reverse transcription kit (Takara, Osaka, Japan). The primer sequences are shown in Supplementary Table 1. qRT-PCR was performed using an ABI Prism 7000 sequence detection system. The Ct values for ENTPD2 mRNA were normalized to those for GAPDH mRNA, which was used as an internal control. The 2^−ΔΔCt^ method was applied to calculate the relative mRNA expression level.

### Western blotting

To obtain total cell lysates and exosome lysates, cells and exosomes were lysed with RIPA buffer (Beyotime, Nanjing, China) containing protease inhibitors (KeyGEN, Nanjing, China). The lysates were centrifuged at 13,000 × g and 4 °C for 15 min. The supernatant fractions were collected and subjected to western blot analysis.

To obtain nuclear and cytosolic lysates, cells were incubated with cold lysis buffer A (10 mM HEPES-NaOH, 10 mM KCl, 1.5 mM MgCl_2_, 0.5 mM dithiothreitol and 0.2 mM phenylmethylsulfonyl fluoride) for 20 min. The samples were then centrifuged at 12,000 × g and 4 °C for 20 min, after which the cytosolic extracts (supernatant fractions) were collected. The nuclear pellets were resuspended in cold lysis buffer B (20 mM HEPES-NaOH, 420 mM NaCl, 1.5 mM MgCl_2_, 0.2 mM EDTA, 0.5 mM dithiothreitol, 2.5 mg/ml leupeptin, 0.2 mM phenylmethylsulfonyl fluoride, 2.5 mg/ml aprotinin, and 25% glycerol) for 30 min. The cytosolic and nuclear extracts were subjected to western blot analysis.

Samples containing equal amounts of protein were separated by SDS‒PAGE, and the separated proteins were transferred to polyvinylidene fluoride membranes (Merck Millipore, Darmstadt, Germany). Then, the membranes were incubated first with the corresponding primary antibody and then with HRP-conjugated goat anti-rabbit IgG or goat anti-mouse IgG as the secondary antibody (Thermo Scientific, Waltham, USA). The antibodies used are listed in Supplementary Table 2. Equal protein loading in the western blot analysis was confirmed with GAPDH or β-actin as the loading control. GAPDH and Lamin B1 were used as markers for the cytosolic and nuclear extracts, respectively. ImageJ software version 5.2.5 was used for densitometric analysis of the bands.

### Cell proliferation and colony formation assays

Cell proliferation was examined using a Cell Counting Kit-8 (KeyGEN, Nanjing, China). Cells were seeded in 96-well plates (2 × 10^3^ cells/well) and further cultured under normal culture conditions for 24, 48 and 72 h. Cell viability was determined according to the manufacturer’s instructions. Colon cancer cells (1000 cells/well) from each group were seeded in six-well plates. The culture medium was changed every 3 days, and the cells were cultured for 14 days and fixed when macroscopically visible clones were observed. The cells were stained with crystal violet for 20 min, and images of the macroscopic clones were acquired.

### Animal studies

Male C57BL/6 mice and male BALB/c athymic nude mice (4–5 weeks) were purchased from the Model Animal Research Center of Nanjing University. All the animal studies were approved by the Animal Ethical and Welfare Committee of Sun Yat-sen University (IACUC-2020090401, IACUC-2022071301).

For the subcutaneous tumor experiments, a total of 3 × 10^5^ cells (MC38-Vector or MC38-ENTPD2) were injected into the dorsal surface of mice (BALB/c or C57BL/6). To study the effect of exosomal ENTPD2 on colon cancer, MC38 cells (3 × 10^5^) were injected into the dorsal surface of C57BL/6 mice on Day 0, exosomes (15 µg) from MC38-ENTPD2 (abbreviated as Exo^PD2−high^) and MC38-Vector (Exo^PD2−LOW^) cells were injected into the mice every 3 days starting on Day 3. For the CD8^+^ T-cell depletion experiments, C57BL/6 mice were intraperitoneally injected with 200 µg of anti-mouse CD8a InVivoMAb (BioXCell, USA) every 4 days starting on day − 1, and MC38 cells (3 × 10^5^) were injected into the dorsal surface of the mice on Day 0. Then, 15 µg of exosomes (Exo^PD2−high^ or Exo^PD2−LOW^) were injected into the tumors every 3 days starting on Day 3. To study the effect of A2AR antagonists on colon cancer, MC38 cells (3 × 10^5^) were injected into the dorsal surface of C57BL/6 mice on Day 0, 15 µg of exosomes (Exo^PD2−high^ or Exo^PD2−LOW^) were injected into the mice every 3 days starting on Day 3. Then, ZM241385 (0.3 µg/mouse) was administered via intraperitoneal injection every 2 days starting on Day 5. The tumors were measured using a digital calliper, and the volume was calculated using the following formula: π/6 × length × width^2^. The mice were sacrificed when the tumors reached 1.5 cm in diameter. tumor tissues harvested from C57BL/6 mice were embedded in normal paraffin and used for IHC analysis. Other mouse tumor tissues were dissociated into single-cell suspensions using a gentleMACS Dissociator (Miltenyi Biotech, Bergisch Gladbach, Germany) according to the manufacturer’s instructions. Single-cell suspensions were subjected to flow cytometric and RNA-seq analyses.

### Immunohistochemical staining and double-labelling immunofluorescence

IHC analysis was conducted as follows. Paraffin sections were dewaxed with xylene and rinsed through a graded ethanol series. Antigen retrieval was performed by boiling in 0.01 M citrate buffer for 25 min. The sections were stained with primary antibodies at 4 °C overnight and then incubated with biotinylated secondary antibodies. Positively stained cells in 5 randomly selected fields were analysed, and manual scoring of the staining intensity, staining location, and types of stained cells was completed by two independent observers.

The equivalent double-labelling immunofluorescence assay was conducted to detect the expression of CD8 and ENTPD2. We incubated the samples first with a rabbit anti-ENTPD2 primary antibody (1:500) and then with the corresponding Cyanine3-conjugated anti-rabbit secondary antibody (Invitrogen, Carlsbad, USA) (1:500). The secondary antibody used was affinity-purified biotinylated goat anti-rabbit IgG. We added the corresponding fluorescein tyramide signal amplification system (TSA) dye. After the samples were subjected to antigen retrieval in a microwave, they were incubated first with an anti-CD8 antibody (1:500) and then with the corresponding FITC-conjugated anti-rabbit secondary antibody (Invitrogen, Carlsbad, USA) (1:500). The primary antibody was replaced with PBS for the negative controls. In the last step, 4,6-diamidino-2-phenylindole (DAPI) was used to stain nuclei. For the evaluation of ENTPD2 expression by immunofluorescence analysis, the mean fluorescence intensity (MFI) was determined using ImageJ 5.2.5 software. All antibodies used are listed in Supplementary Table 2.

### RNA extraction, library construction, RNA sequencing and differential gene expression analysis

Total RNA was extracted from CD8^+^ T cells isolated from mouse tumor tissues using TRIzol reagent (Thermo Scientific, Waltham, USA) following the manufacturer’s instructions. The total RNA quantity and purity were analysed by a Bioanalyzer 2100 and a RNA 6000 Nano LabChip Kit (Agilent Technologies, Palo Alto, USA), and high-quality RNA samples from the MC38-Vector group and MC38-ENTPD2 group were used to construct a library for RNA-seq. Library construction and RNA sequencing were conducted by LC Sciences (Hangzhou, China). After total RNA was extracted, mRNA was purified from total RNA using Dynabeads Oligo (Thermo Scientific, Waltham, USA) with two rounds of purification. Following purification, the mRNA was fragmented into short pieces using divalent cations at elevated temperature. Then, the cleaved RNA fragments were reverse transcribed to cDNA by SuperScript™ II Reverse Transcriptase (Invitrogen, Carlsbad, USA) and were subsequently used to synthesize U-labelled second-strand DNA in a reaction mixture containing *E. coli* DNA polymerase I, RNase H and dUTP Solution (Thermo Scientific, Waltham, USA). There were two biological replicates per group, and the average insert size in the final cDNA library was 300 bp (± 50 bp). Finally, we performed 2 × 150 bp paired-end sequencing (PE150) on the Illumina NovaSeq™ 6000 platform following the vendor’s recommended protocol.

Differential expression between the MC38-Vector group and the MC38-ENTPD2 group was analysed using DESeq2 software. Genes with a false discovery rate (FDR) < 0.05 and absolute fold change ≥ 2 were considered differentially expressed genes (DEGs). The differential expression of selected genes was examined. For heatmap generation, Z scores were calculated from TPM values.

### Isolation of exosomes and preparation of conditioned medium

Exosomes were isolated by differential centrifugation of conditioned medium collected from colon cancer cell lines. The cells were grown in their respective conditioned media to 70% confluence and then cultured in FBS-free medium for 48 h. The supernatants were centrifuged first at 300 × g for 10 min at 4 °C and then at 2,000 × g for 20 min at 4 °C to remove dead cells. The resulting supernatants were filtered through a 0.22 μm mesh to remove large vesicles. Then, the supernatants obtained after filtering were centrifuged at 180,000 × g for 120 min at 4 °C. The resulting final exosome pellets were resuspended in PBS. Exosomes were isolated from serum samples using the ExoQuick Exosome Precipitation Solution Kit (System Biosciences, Mountain View, USA). Briefly, 250 µL of serum was mixed with 63 µL of ExoQuick solution, and the mixture was then incubated for 30 min at 4 °C. The serum/ExoQuick mixture was then centrifuged at 1,500 × g for 30 min at 4 °C, and the pellet remaining after removing the supernatant was resuspended in 300 µL of PBS.

Colon cancer cells were seeded in six-well plates to 70% confluency and then cultured in serum-free medium for 48 h. To prepare the conditioned medium (CM), serum-free medium was collected and centrifuged first at 300 × g for 10 min at 4 °C and then at 2,000 × g for 20 min, after which the supernatant was collected as the CM. Then, the CM was collected and centrifuged at 180,000 × g for 120 min, and the supernatant was collected as exosome-depleted conditioned medium (CM-Exosome). Exosomes isolated from the same number of colon cancer cells were resuspended in 100 µL of PBS. CM and CM-Exosome were concentrated by filtration through Amicon Ultra-15 mL centrifugal filters (Millipore, Billerica, USA) according to the recommended protocol.

### Transmission electron microscopy and NanoTracker analysis of exosomes

The morphology of the exosomes was evaluated via transmission electron microscopy (TEM), and the particle distribution and size were evaluated via dynamic light scattering (Malvern Instruments, Malvern, UK). For TEM, exosomes were placed on a transmission electron microscopy grid, and the excess solution was removed by blotting with filter paper. The exosomes were negatively stained with uranyl acetate at room temperature. The exosomes were then observed using a transmission electron microscope (Hitachi, Tokyo, Japan). For nanoparticle tracking analysis, exosomes were analysed using the NanoSight® software program. The results showed the particle size distribution vs. the intensity (percent).

### Measurement of ATP hydrolytic activity and adenosine levels

To measure ATP consumption in colon cancer cell supernatants, 4 × 10^5^ colon cancer cells were incubated in 500 µL of Opti-MEM in the presence of 150 µM ATP (Sigma, Saint Louis, USA) for 2 h. To explore the distribution of ATPase activity, 100 µL each of CM, CM-Exosome, and Exosome were reacted with 50 µM ATP for 1 h. To detect ATP hydrolytic activity in exosomes, exosomes (30 µg/mL) was reacted with 20 µM ATP in 500 µL of PBS for 1 h. Subsequently, the ATP concentrations were quantified using an ATP Assay Kit (Thermo Scientific, Waltham, USA). The measurements were performed according to the manufacturer’s protocol.

To measure adenosine levels in colon cancer cell supernatants, 4 × 10^5^ colon cancer cells were incubated in 500 µL of Opti-MEM in the presence of 150 µM ATP for 2 h. According to the manufacturer’s protocol, the supernatants were detected by fluorometric assay (Cell Biolabs, San Diego, USA).

### Immunoelectron microscopy

Exosomes were washed twice with PBS, fixed with paraformaldehyde, and placed on copper grids. The grids were blocked with bovine serum albumin for 30 min, incubated with a rabbit polyclonal anti-ENTPD2 antibody for 1 h and labelled with a secondary antibody (anti-rabbit antibody conjugated to 10 nm gold grains) for 1.5 h. The grids were visualized via electron microscopy (FEI Tecnai Spirit TEM T12).

### Determination of ATP, 5’-AMP and adenosine concentrations

The concentrations of ATP, 5′-AMP and adenosine in culture medium were measured using high-performance liquid chromatography (HPLC). The samples were analysed using a Shimadzu HPLC system. ATP, 5′-AMP and adenosine standards (Solarbio, Beijing, China) were used for optimization. The abundances of ATP and its metabolites were represented by the area of each analyte peak.

### Isolation of CD8^+^ T cells from peripheral blood

Peripheral blood mononuclear cells (PBMCs) were isolated using a Ficoll gradient procedure from blood samples collected from healthy donors. CD8^+^ T cells were purified from fresh PBMCs using a MACS CD8^+^ T Cell Isolation Kit (Miltenyi Biotech, Bergisch Gladbach, Germany). CD8^+^ T cells were washed with PBS and cultured in RPMI 1640 medium supplemented with 10% FBS (Gibco, Carlsbad, USA) and 100 IU/mL IL2 (R&D Systems, Minneapolis, USA). CD8^+^ T cells were activated by incubation with anti-CD3/CD28 monoclonal antibodies (mAbs) (eBioscience, San Diego, USA).

### In vitro CD8^+^ T cell proliferation and cytotoxicity assays

In vitro culture systems, we needed to simulate the abnormally elevated ATP concentration in the TME, and we used the ATP concentration (10 µM) reported in an article by Zhang et al. [9]. For the proliferation assay, CD8^+^ T cells were labelled with 1.5 µM CFSE (Thermo Scientific, Waltham, USA) according to the manufacturer’s instructions. CFSE-labelled CD8^+^ T cells were plated in round-bottomed 96-well plates (2 × 10^5^ cells/well) and stimulated with an anti-CD3/CD28 mAb. Next, the CD8^+^ T cells were harvested after exposure to exosomes (30 µg/mL) in the presence of ATP for 48 h and were then analysed by flow cytometry. The concentrations of ATP were determined based on established protocols.

For the cytotoxicity assay, CD8^+^ T cells (2 × 10^5^ cells/well) in a 96-well plate were activated by the addition of an anti-CD3/CD28 mAb and cultured with exosomes (30 µg/mL) in the presence of ATP for 48 h. Subsequently, Granzyme B, TNF-α and IFN-γ expression in CD8^+^ T cells was measured by flow cytometry.

### Flow cytometry

Exosomes were identified using a BD flow cytometer (Accuri C6), and immunophenotyping was performed using two flow cytometers (BD LSR2 and CytoFLEX LX). For cell surface staining (CD3, CD8, PD-1 and CD44), cells were washed with PBS and incubated for 30 min at 4 °C with fluorochrome-conjugated antibodies. For intracellular staining (Granzyme B, TNF-α and IFN-γ), cells were stained with antibodies against cell surface markers and were then fixed and permeabilized with a fixation/permeabilization solution (eBioscience, San Diego, USA) for 20 min at room temperature. After that, the cells were washed with PBS again and incubated for 30 min at room temperature with fluorochrome-conjugated antibodies.

All fluorochrome-conjugated antibodies used for flow cytometric analysis are listed in Supplementary Table 3. All data were analysed using FlowJo 10.0 software. The gating strategy used for analysis of CD8^+^ T cells is shown in Figure S1.

### Statistical analysis

All the statistical analyses were performed using GraphPad Prism V.8 or SPSS 16.0 software. The significance of differences between two independent groups was determined by two-tailed Student’s *t* test or the *χ*^*2*^ test. The significance of differences among multiple groups was determined by one-way ANOVA followed by Tukey’s multiple comparison test. Survival was estimated by Kaplan–Meier analysis, and differences in survival were evaluated by the log-rank test. *P* < 0.05 was considered to indicate a significant difference.

## Results

### ENTPD2 is upregulated in human colon cancer tissues, and ENTPD2 expression indicates a poor prognosis

**Fig. 1:**
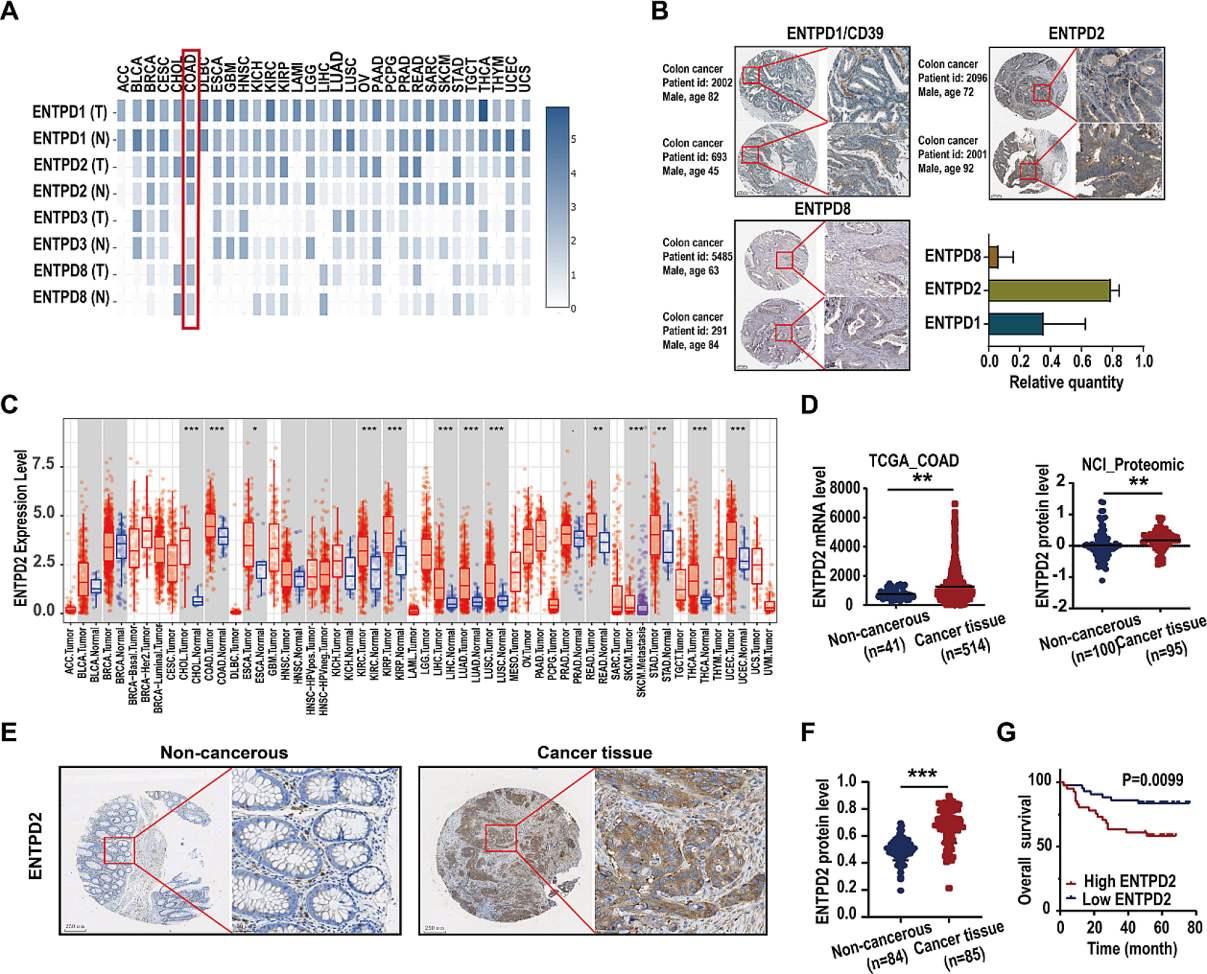
ENTPD2 is frequently overexpressed in human colon cancer cells, and ENTPD2 overexpression indicates a poor prognosis. **A** The expression levels of extracellular ENTPDase family members (CD39, ENTPD2, ENTPD3 and ENTPD8) in various tissues in the GEPIA2 database. **B** CD39, ENTPD2 and ENTPD8 protein expression in 23 colon cancer tissues in the Human Protein Atlas database. **C** Human ENTPD2 expression levels in different cancer types and corresponding normal tissues according to the TIMER2 resource. **D** Relative ENTPD2 mRNA expression levels in 514 colon cancer tissues and 41 noncancerous tissues in a TCGA dataset. Protein expression of ENTPD2 in 95 colon cancer tissues and 100 noncancerous tissues according to NCI proteomics data. **E** Representative IHC staining of ENTPD2 in colon cancer tissues and noncancerous tissues in human tissue microarray. **F** ENTPD2 protein expression in 85 colon cancer tissues and 84 noncancerous tissues, as evaluated by IHC staining of a tissue microarray. **G** Kaplan–Meier analysis of overall survival for 85 colon cancer patients stratified according to the ENTPD2 expression level. Statistical analysis was performed using Pearson correlation analysis. **D, F** Student’s *t* test was used for statistical analysis; ^**^P < 0.01, ^***^P < 0.001

Among the extracellular ENTPDases (CD39, ENTPD2, ENTPD3 and ENTPD8), ENTPD2 had the highest expression in colon tumor tissues, according to analysis of data in GEPIA2 (Fig. 1A). We next examined extracellular ENTPDase protein expression in human colon cancer tissues using the Human Protein Atlas. ENTPD3 was not included because of missing data on its protein expression levels. In 23 colon cancer tissues, the ENTPD2 level was higher than those of CD39 and ENTPD8 (Fig. 1B). Therefore, we designated ENTPD2 as our target protein. Analysis via the TIMER2 resource revealed significant ENTPD2 overexpression in tissues of multiple human tumors, including colon adenocarcinoma (COAD), compared to the paired normal tissues (Fig. 1C). The Cancer Genome Atlas (TCGA) dataset contained data for 514 colon cancer tissues and 41 noncancerous tissues, and in this dataset, ENTPD2 mRNA expression was strongly elevated in human colon cancer tissues (*P* < 0.05, Fig. 1D). Similarly, ENTPD2 protein expression was significantly greater in 95 colon cancer tissues than in 100 noncancerous tissues in the analysed NCI proteomics dataset (*P* < 0.05, Fig. 1D).

Tissue microarray analysis showed that ENTPD2 was expressed primarily in cancer cells and had a distinctive membrane association (Fig. 1E). The ENTPD2 level was significantly higher in colon cancer tissue (*n* = 85) samples than in noncancerous tissue samples (*n* = 84) (*P* < 0.05, Fig. 1F). Based on the ENTPD2 protein expression threshold, we used the tissue microarray data from 85 patient samples to divide the corresponding patients into a high-expression group (*n* = 42) and a low-expression group *(n* = 43). We demonstrated significant associations between the ENTPD2 level and certain clinical progression-related parameters, namely, TNM stage, T stage, and N stage (*P* < 0.05). No correlations between ENTPD2 expression and patient age, sex, or M stage were found (Supplementary Table 4). Figure 1G presents survival curves showing the correlation between ENTPD2 expression and colon cancer patient prognosis. Kaplan–Meier survival analysis suggested that patients with high ENTPD2 expression had considerably poorer overall survival (OS) than did those with low ENTPD2 expression (*P* < 0.05, Fig. 1G). Collectively, this evidence indicates that ENTPD2 but not the other ENTPDase family members plays an essential role in colon cancer progression.

### ENTPD2 suppresses the function of CD8^+^ T cells in the colon cancer TME

**Fig. 2:**
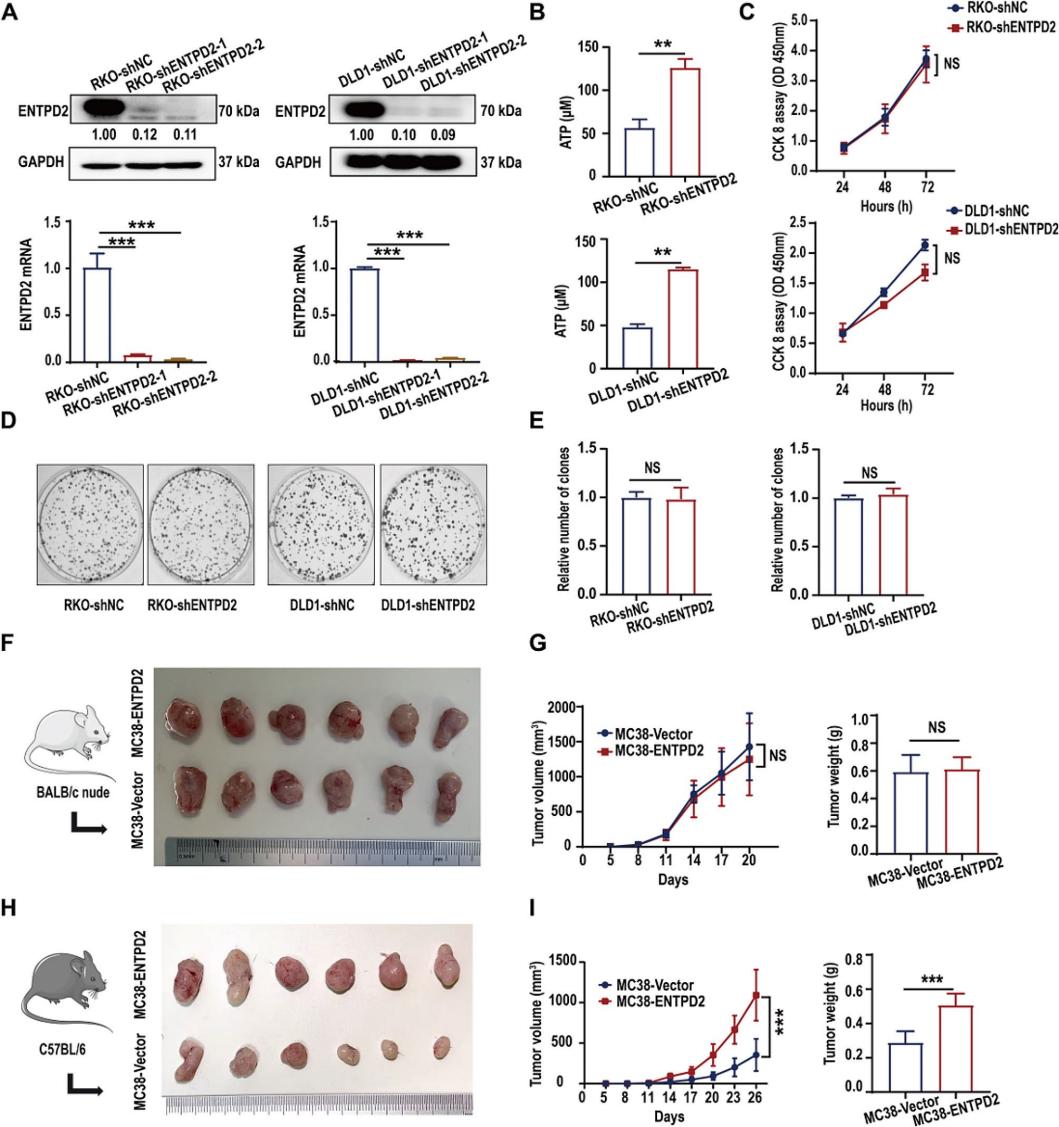
Effect of ENTPD2 on tumor growth in immunodeficient and immunocompetent mouse models. **A** The ENTPD2 knockdown efficiency in RKO and DLD1 cells was validated by qRT‒PCR and western blotting. **B** ATP hydrolytic activity was analysed in the culture medium from RKO and DLD1 cells following treatment with 150 µM ATP for 2 h. **C** CCK-8 assay of cell viability in RKO and DLD1 cells. Cell viability was determined at 24, 48 and 72 h. **D-E** Representative images and quantitative data from colony formation assays in RKO and DLD1 cells. **F** Representative images of tumors from BALB/c nude mice (*n* = 6 mice in each group). **G** Average growth curves and weights of subcutaneous tumors in immunodeficient mice (BALB/c) after inoculation with 3 × 10^5^ MC38-Vector or MC38-ENTPD2 cells. **H** Representative images of tumors from C57BL/6 mice (*n* = 6 mice in each group). **I** Average growth curves and weights of subcutaneous tumors in immunocompetent mice (C57BL/6) after inoculation with 3 × 10^5^ MC38-Vector or MC38-ENTPD2 cells. **A, B, E, G, I** Student’s *t* test was used for statistical analysis; **C, G, I** Two-way ANOVA (with Tukey’s multiple comparison test) was used for statistical analysis; ^**^P < 0.01, ^***^P < 0.001, NS: not significant

We examined the mRNA expression of extracellular ENTPDase family members (CD39, ENTPD2, ENTPD3 and ENTPD8) in RKO cells and found that ENTPD2 was the most highly expressed, while ENTPD8 was only weakly expressed (Figure S2). Transfection of ENTPD2-siRNA significantly decreased ATP hydrolysis in RKO cells, but transfection of ENTPD8-siRNA had little effect on this activity (Figure S2). We therefore evaluated the potential physiological role of ENTPD2 in colon cancer cells. The ATP hydrolysis assay revealed that ENTPD2 knockdown (RKO-shENTPD2 and DLD1-shENTPD2 cells) severely impaired the ability of colon cancer cells to degrade extracellular ATP (*P* < 0.05, Fig. 2A-B). CCK-8 assays showed that ENTPD2 knockdown did not affect the proliferation of RKO or DLD1 cells (Fig. 2C), while colony formation assays showed similar results (Fig. 2D-E). The above results indicate that ENTPD2 does not directly affect the in vitro proliferation ability of colon cancer cells. We further selected the MC38 colon cancer cell line derived from C57BL/6 mice and investigated the effects of ENTPD2 on MC38 cells in vivo (Figure S3). Studies in the immunodeficient mouse model showed that the tumor growth curve and tumor size did not differ significantly between the MC38-ENTPD2 and MC38-Vector groups (*P* > 0.05, Fig. 2F-G). Notably, MC38-ENTPD2 tumors had greater volumes and weights than MC38-Vector tumors in immunocompetent mice (*P* < 0.05, Fig. 2H-I). Collectively, this evidence revealed that ENTPD2 did not directly influence colon cancer cells and that ENTPD2 may promote tumor growth in immunocompetent mice by affecting the tumor microenvironment.

**Fig. 3:**
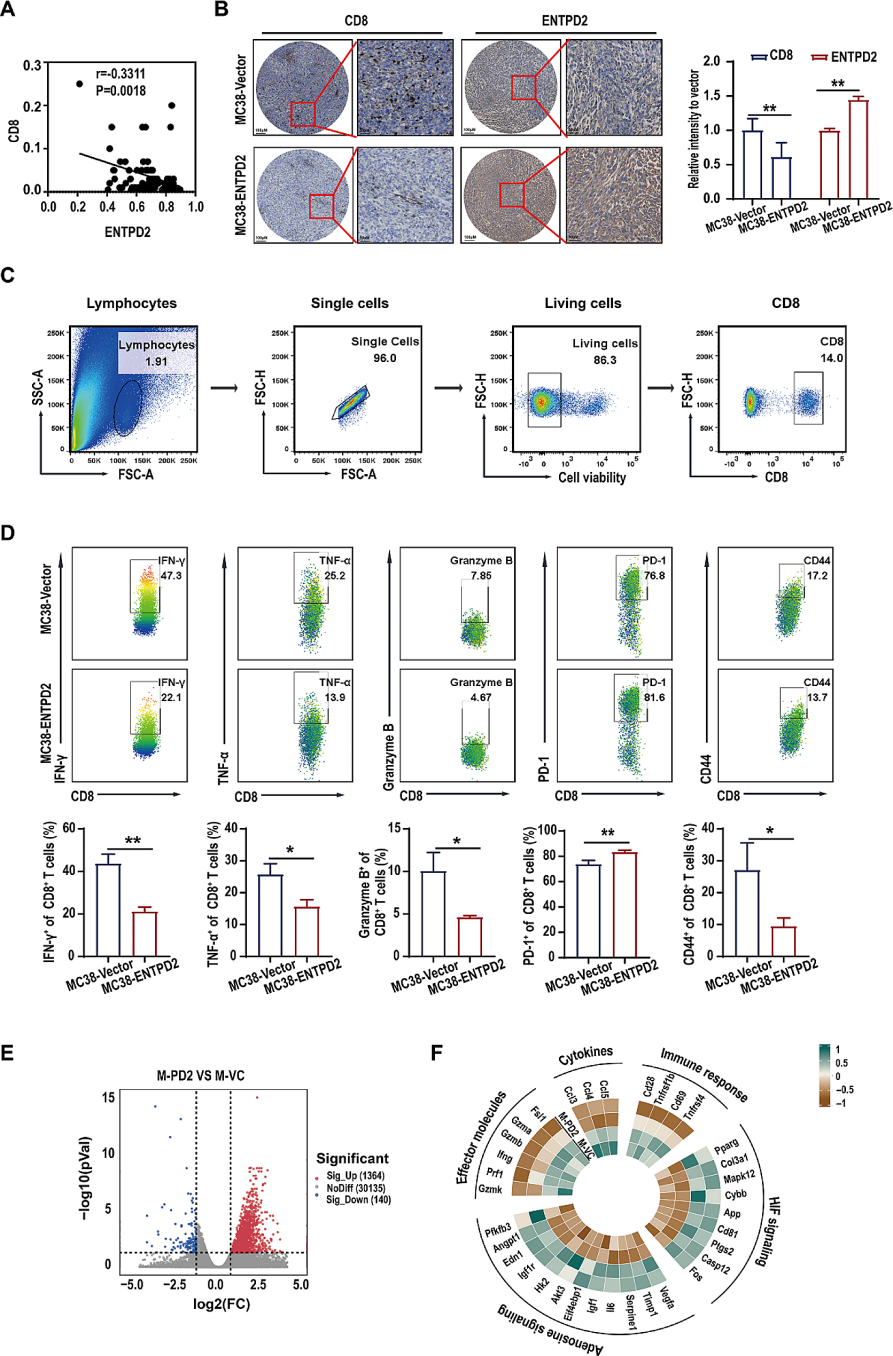
ENTPD2 suppresses the function of CD8^+^ T cells in the colon cancer TME. **A** A negative correlation between the CD8^+^ T-cell and intratumoral ENTPD2 level was detected in 85 human colon cancer tissues. **B** Immunohistochemical staining for ENTPD2 and CD8 in sections of mouse tumor tissues from the MC38-Vector group and MC38-ENTPD2 group. **C** The gating strategy used to identify the phenotype of CD8^+^ T cells isolated from subcutaneous tumor tissues. **D** Tumors from the MC38-Vector group and MC38-ENTPD2 group were dissociated, and the expression patterns of IFN-γ, TNF-α, Granzyme B, PD-1 and CD44 in CD8^+^ T cells were characterized by flow cytometry. **E** Volcano plot of genes whose expression was significantly upregulated (red), significantly downregulated (blue), or remained unchanged (gray) in CD8^+^ T cells from the MC38-ENTPD2 (M-PD2) and MC38-Vector (M-VC) groups. **F** Representative heatmap of the Z scores for selected genes in CD8^+^ T cells from the MC38-ENTPD2 (M-PD2) and MC38-Vector (M-VC) groups. **B, D** Student’s *t* test was used for statistical analysis; ^*^P < 0.05, ^**^P < 0.01

Tumor-infiltrating immune cells are crucial for the TME and have a considerable impact on tumor progression [10]. Therefore, we investigated the relationship between ENTPD2 expression and immune cell infiltration in colon cancer. Analysis via the TISIDB portal revealed that the ENTPD2 expression level was negatively correlated with the infiltration of immune cells, including activated CD8^+^ T cells (Act_CD8, *P* < 0.001), effector memory CD8^+^ T cells (Tem_CD8, *P* < 0.001), activated CD4^+^ T cells (Act_CD4, *P* < 0.001), neutrophils (*P* < 0.001), macrophages (*P* < 0.001), MDSCs (*P* < 0.001) and activated B cells (Act_B, *P* < 0.01) (Figure S4). Similar findings were obtained via TIMER analysis, indicating significant negative correlations between the expression of ENTPD2 and the infiltration of CD8^+^ T cells, macrophages and neutrophils in colon cancer (*P* < 0.05, Figure S4). Since cytotoxic CD8^+^ T cells are crucial for adaptive immune resistance and play a crucial role in the antitumor immune response, we quantified CD8^+^ T-cell infiltration in a human colon cancer tissue microarray. Pearson correlation analysis revealed a negative correlation between the quantity of CD8^+^ T cells and intratumoral ENTPD2 expression (*P* < 0.05, Fig. 3A). In addition, immunohistochemical staining of C57BL/6 tumor tissues suggested that the quantity of CD8^+^ T cells in the MC38-ENTPD2 group was significantly lower than that in the MC38-Vector group (*P* < 0.05, Fig. 3B); thus, it is rational to hypothesize that ENTPD2 can affect the CD8^+^ T-cell response.

Using flow cytometry, we characterized the phenotype of CD8^+^ T cells in the tumor tissues of C57BL/6 mice (Fig. 3C). CD44 is a valuable marker for highly proliferating CD8^+^ T cells [11]. We demonstrated that the proportion of CD44^+^ T cells among CD8^+^ T cells was substantially lower in the MC38-ENTPD2 group than in the MC38-Vector group (*P* < 0.05, Fig. 3D). Among the major roles of activated CD8^+^ T cells are cytotoxic cytokine (IFN-γ and TNF-α) and cytolytic molecule (Granzyme B) production [12]. Our results revealed that the numbers of IFN-γ^+^CD8^+^, TNF-α^+^CD8^+^, and Granzyme B^+^CD8^+^ T cells were strongly decreased in the MC38-ENTPD2 group (*P* < 0.05, Fig. 3D). Programmed death 1 receptor (PD-1) is an immune checkpoint molecule expressed on chronically stimulated T cells that leads to reduced production of cytotoxic cytokines [13]. PD-1 was upregulated on CD8^+^ T cells in the MC38-ENTPD2 group compared with the MC38-Vector group (*P* < 0.05, Fig. 3D). Collectively, these results suggested that ENTPD2 overexpression inhibited CD8^+^ T-cell activation while reducing the cytotoxic activity of CD8^+^ T cells.

RNA-seq analysis of CD8^+^ T cells isolated from tumor tissues in immunocompetent C57BL/6 mice revealed a total of 1504 differentially expressed genes. Among these genes, 1364 were upregulated, and 140 were downregulated (Fig. 3E). In the MC38-ENTPD2 (M-PD2) group, several genes were found to be significantly upregulated. These included genes involved in HIF signalling (*Pparg, Col3a1, Mapk12, Cybb, App, Cd81, Ptgs2, Casp12*, and *Fos*), as well as adenosine signalling (*Vegfa, Timp1, Serpine1, Il6, Igf1, Eif4ebp1, Akt3, Hk2, Igf1r, Edn1, Angpt1*, and *Pfkfb3*). On the other hand, the downregulated genes were related to cytokines (*Ccl3, Ccl4*, and *Ccl5*), T-cell effector molecules (*Gzmk, Gzmb, Gzma, Ifng, Prf1, Fasl*, and *Tnfrsf1b*), and the immune response (*Cd28, Cd69*, and *Tnfrsf4*) (Fig. 3F). Collectively, our findings support the idea that ENTPD2 overexpression accelerates cancer development in C57BL/6 mice via inhibition of CD8^+^ T-cell function.

### ENTPD2 is localized primarily in exosomes, and exosomal ENTPD2 inhibits CD8^+^ T-cell function

**Fig. 4:**
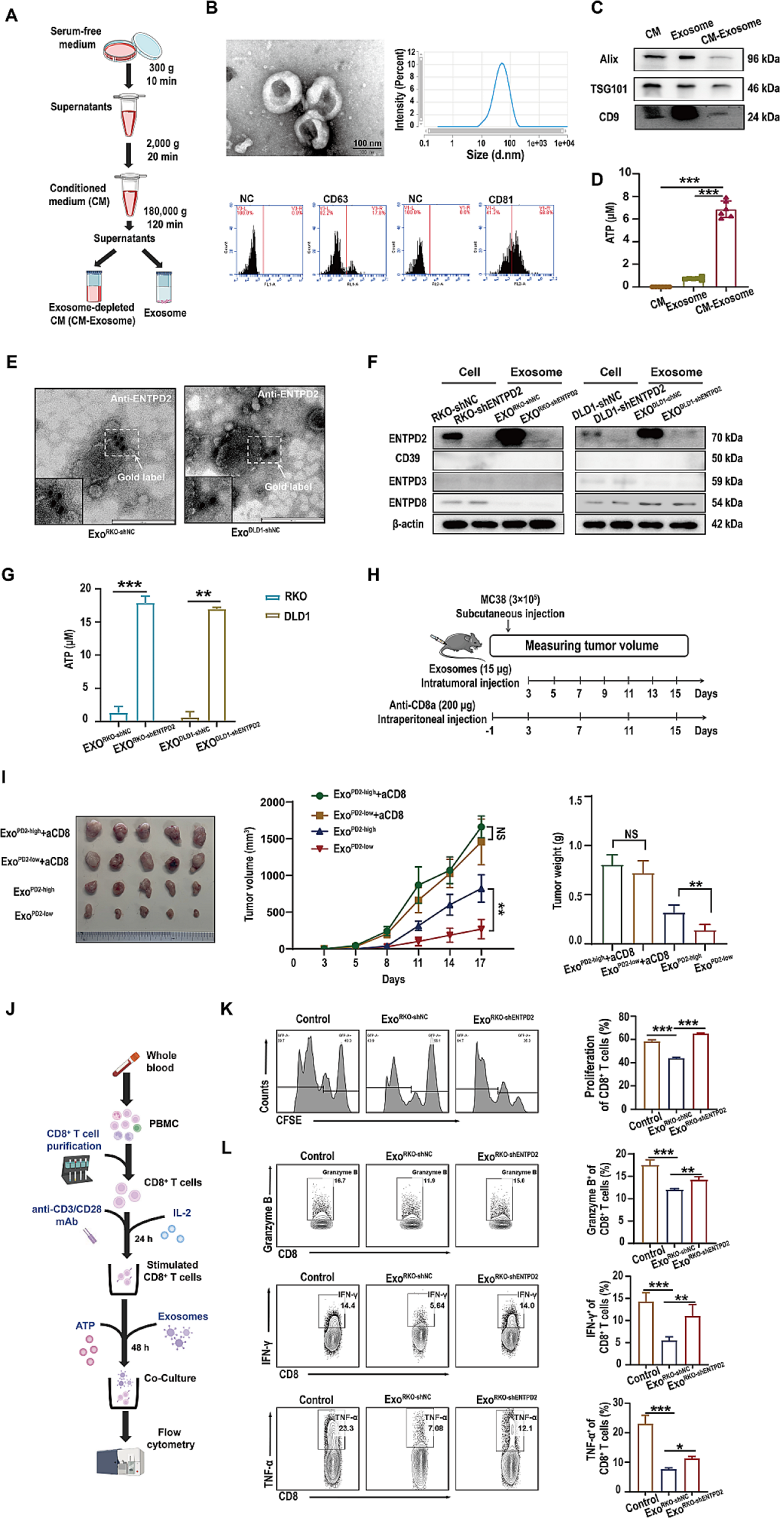
ENTPD2 is primarily carried by exosomes derived from colon cancer cells and inhibits CD8^+^ T-cell function. **A** Experimental scheme for preparing CM, Exosome and CM-Exosome. **B** Exosomes purified from RKO cells were identified by transmission electron microscopy, NanoTracker analysis and flow cytometry. **C** Western blot analysis of exosome markers (Alix, TSG101 and CD9) in the CM, Exosome and CM-Exosome groups. **D** ATP hydrolysis activity in CM, Exosome and CM-Exosome was measured in vitro. **E** Exosome samples (Exo^RKO−shNC^ and Exo^DLD1−shNC^) were subjected to immunogold staining with an anti-ENTPD2 antibody (IgG) and a 10 nm gold grain-conjugated secondary antibody and examined by TEM. **F** Western blot analysis of extracellular ENTPDases (CD39, ENTPD2, ENTPD3 and ENTPD8) in both colon cancer cells and exosomes. The same amounts of exosomal protein were loaded. **G** ATP hydrolysis activity in 30 µg/mL exosomes derived from RKO cells (Exo^RKO−shNC^, Exo^RKO−shENTPD2^) and exosomes derived from DLD1 cells (Exo^DLD1−shNC^, Exo^DLD1−shENTPD2^) was measured in vitro. **H** Schematic of the in vivo subcutaneous tumorigenesis experiment. **I** Representative images, average growth curves and weights of tumors from C57BL/6 mice treated with an anti-CD8a or isotype control antibody followed by treatment with exosomes (Exo^PD2−high^ or Exo^PD2−low^) and tumor challenge with 3 × 10^5^ MC38 cells. **J** Experimental scheme for the coculture of stimulated CD8^+^ T cells and exosomes. **K** Representative histograms showing proliferation data of stimulated CD8^+^ T cells incubated with exosomes from colon cancer cells. Exo^RKO−shNC^ or Exo^RKO−shENTPD2^ (30 µg/mL) were cocultured with human peripheral blood-derived CD8^+^ T cells labelled with CFSE and prestimulated with an anti-CD3/CD28 mAb and IL-2. The percentage of CFSE-stained CD8^+^ T cells was analysed by flow cytometry. **L** Representative contour plots of stimulated CD8^+^ T cells examined for the expression of Granzyme B, IFN-γ and TNF-α after incubation with exosomes from colon cancer cells. Stimulated CD8^+^ T cells were cultured with 30 µg/mL Exo^RKO−shNC^ or Exo^RKO−shENTPD2^ in the presence of ATP. After 48 h, Granzyme B, TNF-α and IFN-γ production by CD8^+^ T cells was analysed by flow cytometry. **D, K, L** One-way ANOVA was used for statistical analysis. **I** Two-way ANOVA (with Tukey’s multiple comparison test) was used for statistical analysis. **G, I** Student’s *t* test was used for statistical analysis; ^*^P < 0.05, ^**^P < 0.01, ^***^P < 0.001, NS: not significant

The above results demonstrate a close correlation between ENTPD2 and the function of CD8^+^ T cells in colon cancer. How does ENTPD2 derived from colon cancer cells exert inhibitory effects on CD8^+^ T cells? We conducted preliminary investigations into the presence of ENTPD2 in colon cancer cells, specifically exploring whether ENTPD2 exists in a soluble form or in exosomes in the extracellular space. Herein, we prepared conditioned medium (CM), CM-Exosome, and Exosome from colon cancer cells (Fig. 4A) to investigate differences in ATP hydrolysis, which is regulated primarily by ENTPD2. TEM and particle size analyses revealed that the vesicular exosome had an average diameter of 56.7 nm (Fig. 4B). Moreover, we detected the classical markers CD63 and CD81 in purified exosomes using flow cytometry, and the results suggested that this isolation protocol was appropriate for subsequent experiments (Fig. 4B). Exosomal markers (Alix, TSG101 and CD9) were ubiquitously expressed in CM and Exosome; however, their expression was relatively low in CM-Exosome (Fig. 4C). After incubating CM, CM-Exosome and Exosome with ATP, we found that the remaining ATP content in CM-Exosome was significantly greater than that in CM and Exosome (*P* < 0.05, Fig. 4D). Collectively, the results of these assays revealed that ATPase expression and activity were strongly elevated in tumor-derived exosomes.

ENTPD2 is a principal ATP hydrolytic enzyme within colon cancer cells, and it primarily localizes within the cell membrane, with its catalytic site oriented towards the extracellular space [14]. Therefore, we examined whether an abundance of ENTPD2 could be detected within exosomes isolated from colon cancer cells. Using immunoelectron microscopy (Fig. 4E), we found that exosomal ENTPD2 has the same membrane topology as cell surface ENTPD2, the domain exposed on the surface of exosomes (Exo^RKO−shNC^ and Exo^DLD1−shNC^). Subsequently, we evaluated exosomes derived from ENTPD2-knockdown colon cancer cells (Exo^RKO−shENTPD2^ and Exo^DLD1−shENTPD2^) and control colon cancer cells (Exo^RKO−shNC^ and Exo^DLD1−shNC^), with the corresponding cell lysates used as controls. We found that ENTPD2 was highly abundant in Exo^RKO−shNC^ and Exo^DLD1−shNC^ but minimally abundant in Exo^RKO−shENTPD2^ and Exo^DLD1−shENTPD2^ (Fig. 4F). In addition to ENTPD2, other extracellular ENTPDases (CD39, ENTPD3 and ENTPD8) also possess ATP hydrolysis activity. Our analysis of CD39, ENTPD3 and ENTPD8 expression in colon cancer cell-derived exosomes revealed relatively low expression in both cell lysates and control cell-derived exosomes (Exo^RKO−shNC^ and Exo^DLD1−shNC^). Moreover, the reduction in CD39, ENTPD3 and ENTPD8 expression remained unaltered by stable ENTPD2 knockdown in Exo^RKO−shENTPD2^ and Exo^DLD1−shENTPD2^ (Fig. 4F). On the other hand, we observed that the concentrations of remaining ATP in Exo^RKO−shENTPD2^ and Exo^DLD1−shENTPD2^ was substantially greater than those in Exo^RKO−shNC^ and Exo^DLD1−shNC^ (*P* < 0.05, Fig. 4G). The aforementioned data revealed that ENTPD2 was abundant in exosomes derived from colon cancer cells. It played a dominant role in ATP hydrolysis in these exosomes.

Exosomes originate from tumor cells and carry a molecular signature that partially reflects the characteristics of the parental tumor cells [15]. To examine the potential role of exosomal ENTPD2 in tumor progression, we injected the Exo^PD2−high^ and Exo^PD2−low^ into C57BL/6 mice. Compared to the Exo^PD2−low^ group and the Control group, the Exo^PD2−high^ group exhibits a significant promotion of tumor growth (*P* < 0.05, Figure S5). As expected, significantly lower expression of CD8^+^ T cells was found in the Exo^PD2−high^ group compared with Exo^PD2−low^ group. In addition, IHC analysis revealed lower expression of neutrophils (MPO) and macrophages (CD68) in the Exo^PD2−high^ group (*P* < 0.05, Figure S5). CD8^+^ T cells are effector cells in tumor immunity, when MC38 cells were implanted into mice lacking CD8^+^ T cells using anti-mouse CD8a antibodies, the tumor-promoting effect of exosomal ENTPD2 was eliminated (*P* < 0.05, Fig. 4H-I). We observed greater tumor infiltration of CD8^+^ T cells in the Exo^PD2−low^ group than in the Exo^PD2−high^ group, and this difference was prevented by treatment with the anti-CD8a antibody (*P* < 0.05, Figure S5).

Next, we analysed the effects of exosomal ENTPD2 on CD8^+^ T cells in vitro by isolating PBMCs from healthy donors (Fig. 4J). Upon activation, naïve CD8^+^ T cells proliferate and differentiate to generate effector CD8^+^ T cells, which, in turn, synthesize copious amounts of cytotoxic effector molecules (Granzyme B and Perforin) and inflammatory cytokines (IFN-γ and TNF-α) that kill tumor cells [16]. Compared with the negative controls, Exo^RKO−shNC^ strongly suppressed CD8^+^ T-cell proliferation (Fig. 4K). However, relative to Exo^RKO−shNC^, Exo^RKO−shENTPD2^ strongly enhanced CD8^+^ T-cell proliferation (Fig. 4K). Moreover, Exo^RKO−shNC^ strongly suppressed CD8^+^ T-cell cytotoxicity, as evidenced by the reduced production of Granzyme B, IFN-γ and TNF-α. Alternately, treatment of CD8^+^ T cells with Exo^RKO−shENTPD2^ effectively abolished the aforementioned effects (Fig. 4L). We noted similar observations in experiments using DLD1 cell-derived exosomes that expressed ENTPD2 (Figure S6). Collectively, both the in vitro and in vivo experimental results indicate that exosomal ENTPD2 derived from colon cancer cells can significantly inhibit the function of CD8^+^ T cells.

### Exosomal ENTPD2 suppresses the functions of CD8^+^ T cells by inhibiting upstream ATP-mediated NFATc1 activation

**Fig. 5:**
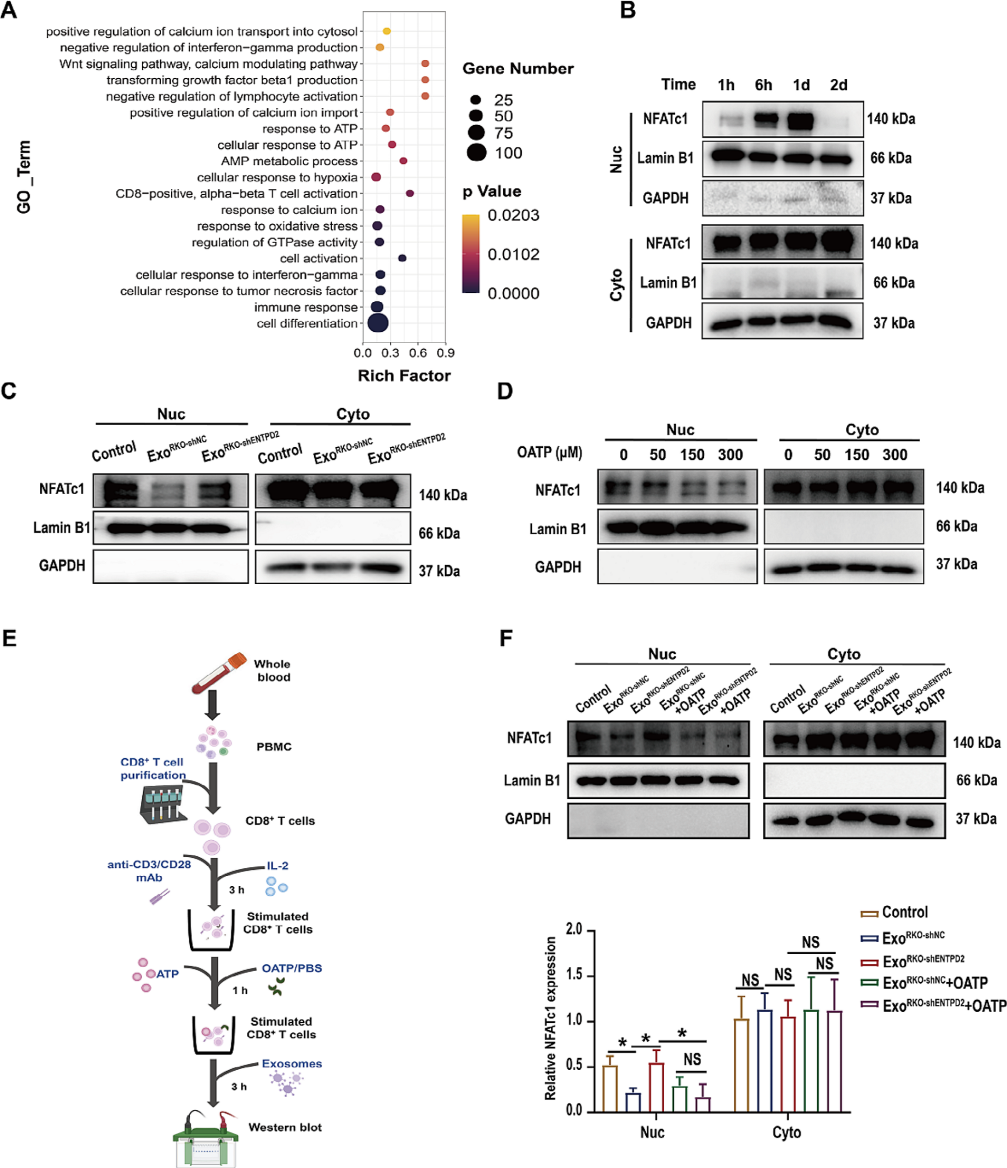
Exosomal ENTPD2 suppresses the functions of CD8^+^ T cells by inhibiting P2  X7R-mediated NFATc1 activation. **A** Biological process analysis results based on the differentially expressed genes in the total CD8^+^ T cell population between the MC38-Vector (M-VC) and MC38-ENTPD2 (M-PD2) groups. The size of the dot represents the number of significantly differentially expressed genes, and a smaller *P* value indicates that the GO term is more significantly enriched. **B** Time course (1 h, 6 h, 1 d, 2 d) of nuclear NFATc1 activation in human CD8^+^ T cells treated with the anti-CD3/CD28 mAb. **C** Western blot for analysis of the activation of NFATc1 in stimulated CD8^+^ T cells incubated with exosomes from colon cancer cells. Human CD8^+^ T cells were incubated with 30 µg/mL Exo^RKO−shNC^ or Exo^RKO−shENTPD2^ after prestimulation with an anti-CD3/CD28 mAb and IL-2. **D** Inhibition of anti-CD3/CD28-induced NFATc1 activation and nuclear translocation by the P2 X7R antagonist OATP. CD8^+^ T cells were prestimulated with an anti-CD3/CD28 mAb and then treated with different concentrations of OATP. **E** Experimental scheme for the addition of the P2 X 7R antagonist OATP to the coculture system of CD8^+^ T cells and exosomes. **F** Western blot analysis of NFATc1 activation in stimulated CD8^+^ T cells incubated with exosomes from colon cancer cells in the presence or absence of P2  X7R antagonists. Human CD8^+^ T cells were prestimulated with an anti-CD3/CD28 mAb and then incubated with 30 µg/mL Exo^RKO−shNC^ or Exo^RKO−shENTPD2^ in the presence or absence of OATP (300 µM). **F** One-way ANOVA was used for statistical analysis; ^*^P < 0.05, NS: not significant

Gene Ontology analysis revealed that the differentially expressed genes shown in Fig. 3E are involved in multiple biological processes, including several processes related to Ca^2+^ influx and T-cell activation (Fig. 5A). Ca^2+^ influx-induced NFATc1 activation is essential for T-cell function and adaptive immunity [17], and we explored whether exosomal ENTPD2 affects NFATc1 activation in CD8^+^ T cells. NFATc1 in naïve CD8^+^ T cells is phosphorylated and located in the cytoplasm, but upon stimulation, it is dephosphorylated, activated, and translocated to the nucleus to induce the expression of genes responsible for CD8^+^ T-cell function [17]. Nuclear NFATc1 activation was evident after only 6 h of anti-CD3/CD28 mAb stimulation (Fig. 5B); therefore, we chose 6 h poststimulation as the time point for subcellular localization analysis of NFATc1 in human CD8^+^ T cells. We first investigated the effect of exosomal ENTPD2 on nuclear NFATc1 activation in CD8^+^ T cells. Compared to that in the negative control cells, the NFATc1 abundance in the nuclear fraction was decreased in the Exo^shNC^ groups (Exo^RKO−shNC^ and Exo^DLD1−shNC^) (Fig. 5C and Figure S7). Moreover, the decrease in the NFATc1 abundance in the nuclear fraction in the Exo^shNC^ group was enhanced in the Exo^shENTPD2^ group (Exo^RKO−shENTPD2^ and Exo^DLD1−shENTPD2^) (Fig. 5C and Figure S7), indicating that the ENTPD2-containing exosomes effectively abolished NFATc1 activation. Alternately, the NFATc1 abundance in the cytoplasmic fraction was similar to negative control Exo^shNC^ group and the Exo^shENTPD2^ group. This may be due to the excessive cytoplasmic expression of NFATc1. These data indicate that exosomal ENTPD2 plays a previously unknown role in nuclear NFATc1 activation in stimulated CD8^+^ T cells.

Nuclear translocation of NFATc1 can be mediated by the ATP-sensitive purinergic receptor (P2X7R) [18], and the P2X7R antagonist OATP successfully inhibited nuclear NFATc1 in stimulated CD8^+^ T cells (Fig. 5D). Then, we tested whether P2X7R is involved in modulating exosomal ENTPD2-mediated NFATc1 activation in CD8^+^ T cells (Fig. 5E). Compared with Exo^shNC^ (Exo^RKO−shNC^ and Exo^DLD1−shNC^) group, the Exo^shENTPD2^ (Exo^RKO−shENTPD2^ and Exo^DLD1−shENTPD2^) group showed increased nuclear NFATc1 expression, and this increase was prevented by the blockade of P2 X7R with OATP (*P* < 0.05, Fig. 5F and Figure S7). These findings suggest that exosomal ENTPD2 functions as a crucial inhibitory factor of NFATc1 activation in CD8^+^ T cells within the TME through the suppression of the ATP-P2  X7R pathway.

### Exosomal ENTPD2 suppresses the functions of CD8^+^ T cells by promoting the adenosine-A2AR pathway

**Fig. 6:**
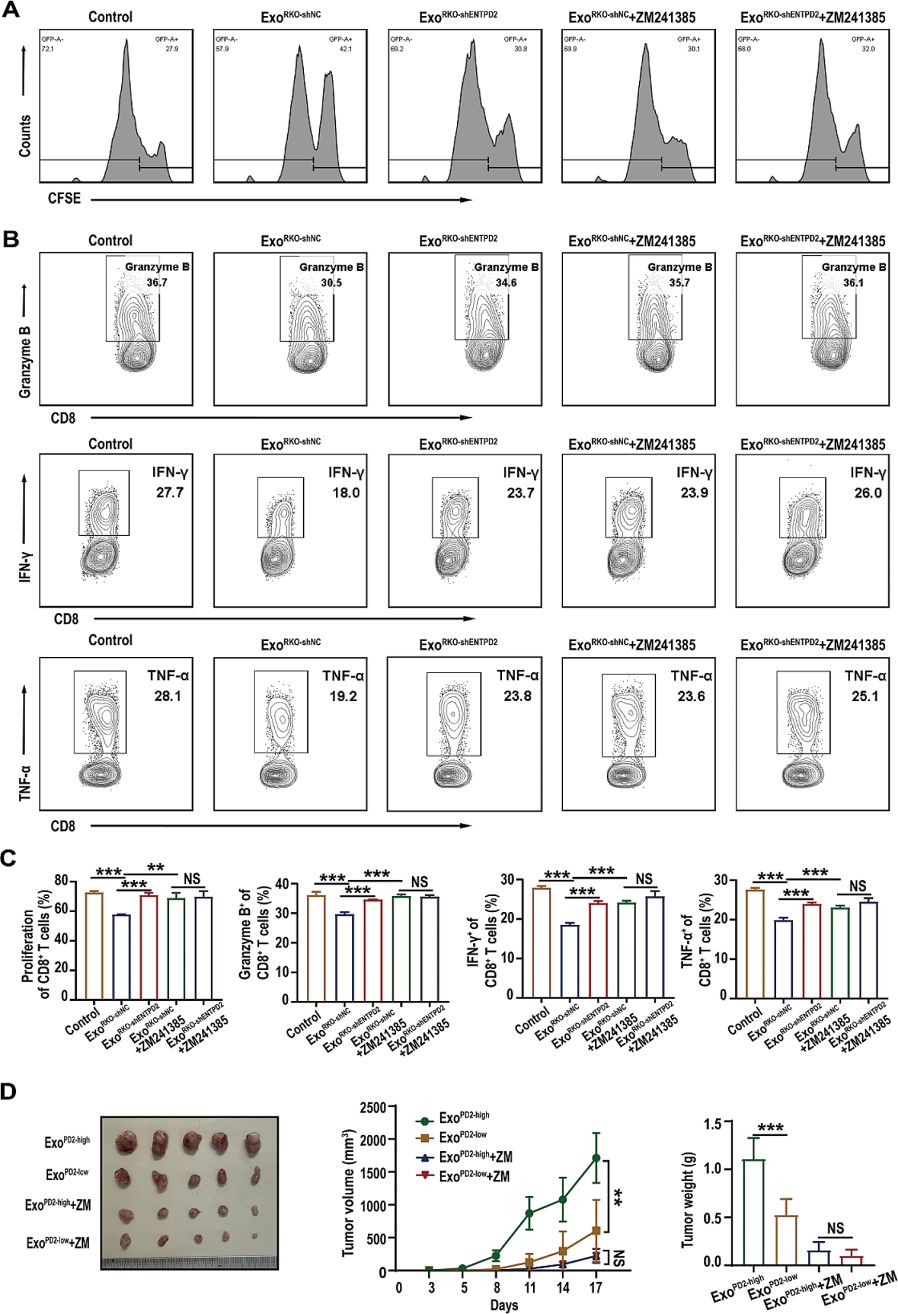
Exosomal ENTPD2 suppresses the functions of CD8^+^ T cells by promoting the adenosine-A2AR pathway. **A** Representative proliferation histograms of stimulated CD8^+^ T cells incubated with exosomes from colon cancer cells in the presence or absence of ZM241385 (1 µM). Exo^RKO−shNC^ or Exo^RKO−shENTPD2^ (30 µg/mL) were cocultured with human peripheral blood CD8^+^ T cells labelled with CFSE and prestimulated with an anti-CD3/CD28 mAb and IL-2. The percentage of CFSE-stained CD8^+^ T cells was analysed by flow cytometry. **B** Representative contour plots of stimulated CD8^+^ T cells examined for the expression of Granzyme B, IFN-γ and TNF-α after incubation with exosomes from colon cancer cells in the presence or absence of ZM241385 (1 µM). Anti-CD3/CD28 mAb-stimulated CD8^+^ T cells were cultured with 30 µg/mL Exo^RKO−shNC^ or Exo^RKO−shENTPD2^ in the presence or absence of ZM241385. After 48 h, Granzyme B, TNF-α and IFN-γ production by CD8^+^ T cells was analysed by flow cytometry. **C** The percentage of each subpopulation, as determined from three independent experiments, is shown. **D** Representative images, average growth curves and tumor weight of C57BL/6 mice treated with ZM241385 followed by treatment with exosomes (Exo^PD2−high^ or Exo^PD2−low^) and tumor challenge with 3 × 10^5^ MC38 cells. **C, D** One-way ANOVA was used for statistical analysis; **D** Two-way ANOVA (Tukey’s multiple comparisons test) was performed for statistical analysis; ^**^P < 0.01, ^***^P < 0.001, NS: not significant

HPLC analysis revealed that knockdown of ENTPD2 significantly decreased the extracellular concentration of adenosine while increasing the extracellular concentration of ATP in the CM of RKO cells (Figure S8). It was also evident from the adenosine detection experiment that ENTPD2 knockdown in RKO cells inhibited the production of adenosine (Figure S8). As mentioned above, the results of RNA sequencing of mouse intratumoral CD8^+^ T cells showed that ENTPD2 promoted the upregulation of genes related to the adenosine pathway (Fig. 3F). These data suggest that ENTPD2 can promote extracellular adenosine accumulation. Adenosine interacts with extracellular adenosine receptors on various immune cells, exerting potent immunosuppressive effects [19, 20]. Four different adenosine receptors have been reported: A1R, A2AR, A2BR and A3R [20]. A2AR is the predominant adenosine receptor on T cells, and it potently suppresses the function of T cells [20]. We revealed that CFSE-labelled CD8^+^ T cell proliferation in the Exo^RKO−shNC^/Exo^DLD1−shNC^ groups was substantially elevated by treatment with the A2AR antagonist ZM241385 (*P* < 0.05, Fig. 6A and Figure S8). Conversely, we observed no marked difference in CD8^+^ T cell proliferation between the Exo^RKO−shNC^+ZM241385/Exo^DLD1−shNC^+ZM241385 groups and the corresponding Exo^RKO−shENTPD2^+ZM241385/Exo^DLD1−shENTPD2^+ ZM241385 groups (*P* > 0.05, Fig. 6A, C and Figure S8). We also observed significant increases in the levels of cytotoxic cytokines, including granzyme B, IFN-γ, and TNF-α, in the Exo^RKO−shENTPD2^/Exo^DLD1−shENTPD2^ groups compared to the Exo^RKO−shNC^/Exo^DLD1−shNC^ groups. The addition of the A2AR antagonist ZM241385 partially reversed this effect (*P* < 0.05, Fig. 6B, C and Figure S8). In vivo animal models, the use of the A2AR antagonist ZM241385 delayed tumor growth and reduced the difference between the Exo^PD2−high^ and Exo^PD2−low^ groups (*P* < 0.05, Fig. 6D). Collectively, these results indicated that exosomal ENTPD2 suppressed the proliferation and cytotoxicity of CD8^+^ T cells by promoting the activity of the adenosine-A2AR pathway.

### Exosomal ENTPD2 levels correlate with clinicopathological progression and tumor infiltration of CD8^+^ T cells in colon cancer patients

**Fig. 7:**
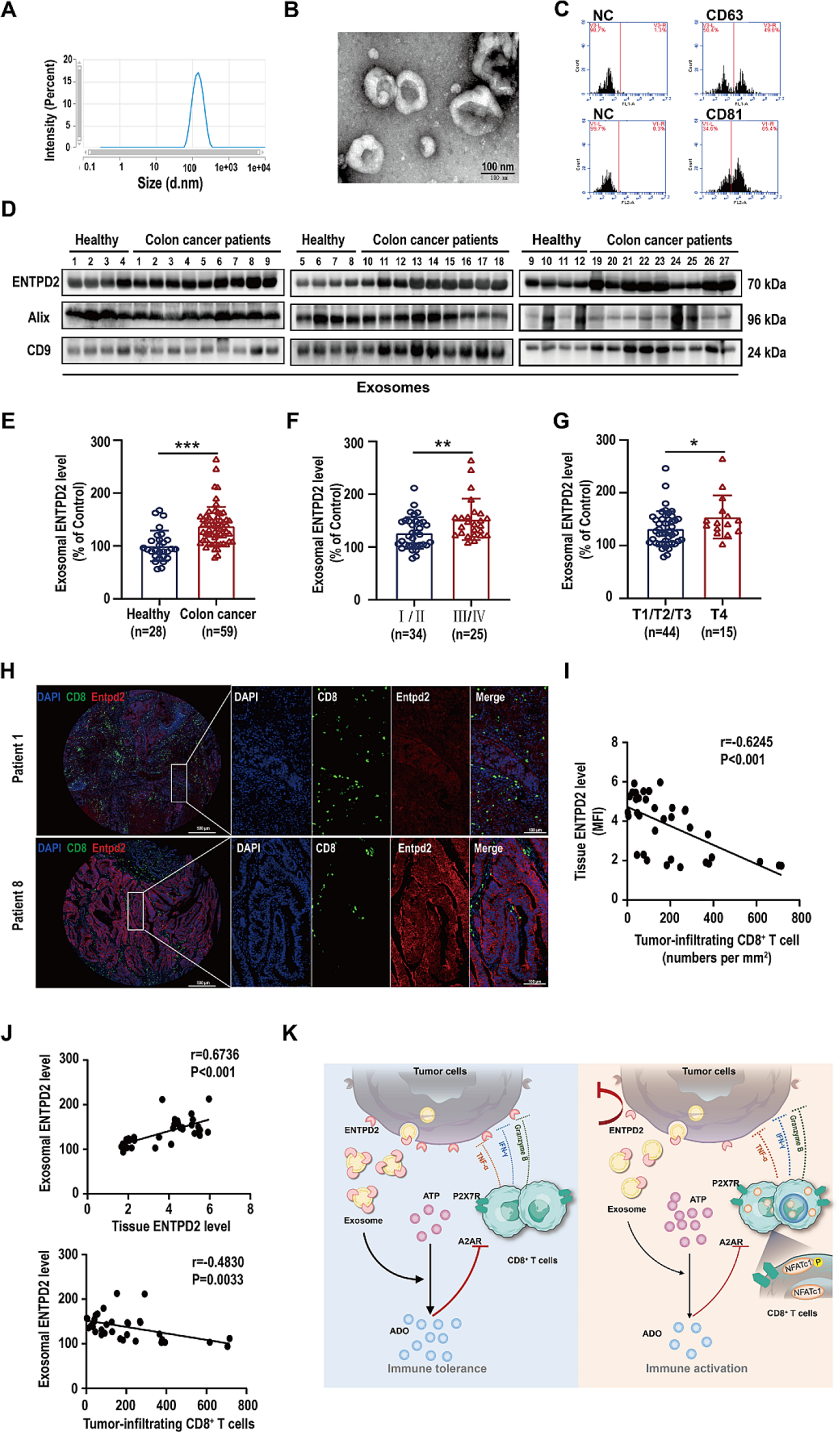
Exosomal ENTPD2 levels correlate with clinicopathological progression and tumor infiltration of CD8^+^ T cells in colon cancer patients. **A** Size distribution of exosomes isolated and purified from the serum of patients with colon cancer (mean diameter 149.8 nm). **B** Representative TEM images of exosomes isolated from the serum of patients with colon cancer. **C** Flow cytometric analysis of the expression of the exosome-enriched proteins CD63/CD81 in exosomes isolated and purified from the serum of patients with colon cancer. **D** Representative western blot showing the abundances of ENTPD2 and typical exosomal markers (Alix and CD9) in exosomes isolated from the serum of colon cancer patients. Exosomes isolated from healthy individuals served as normal controls. **E** Quantitative analysis of exosomal ENTPD2 levels in healthy donors (*n* = 28) and colon cancer patients (*n* = 59). The data are expressed as the percentages of the mean value in the control group (healthy donors). **F-G** Exosomal ENTPD2 levels were higher in patients with advanced-stage colon cancer (stages III and IV, *n* = 25) (**F**) and a high tumor invasion depth (T4, *n* = 15) (**G**). **H** Representative cell staining patterns of CD8 (green) and ENTPD2 (red) in colon cancer tissue samples. Nuclei are pseudocoloured blue. **I** Negative correlation between the tumor-infiltrating CD8^+^ T cell count and the ENTPD2 level in human colon cancer tissues (*n* = 35). **J** The correlations between the abundance of exosomal ENTPD2 in the serum of colon cancer patients (*n* = 35) and the number of tumor-infiltrating CD8^+^ T cells as well as the expression of ENTPD2 in the corresponding tumor tissue. **K** Schematic representation of the role of exosomal ENTPD2 in CD8^+^ T-cell function. **E, F, G** Student’s *t* test was used for statistical analysis; ^*^P < 0.05; ^**^P < 0.01, ^***^P < 0.001

To elucidate the feasibility of ENTPD2 detection in the serum of patients with colon cancer, we isolated and characterized exosomes from the serum of patients with colon cancer. Particle size and TEM analyses confirmed that the exosomal vesicles had an average diameter of 149.8 nm (Fig. 7A, B). Moreover, flow cytometry revealed substantial expression of well-established exosome-specific markers (CD63 and CD81) (Fig. 7C). The typical exosome size, morphology, and protein marker profile suggested the successful isolation of exosomes from human serum. Subsequently, exosomes were isolated from the serum of 59 colon cancer patients, among whom 36 were male (61%) and 23 were female (39%), with a median age of 57 years (range 23–82 years). In addition, we isolated serum exosomes from 28 healthy donors to serve as controls. The clinicopathological and demographic characteristics of the study participants are summarized in Supplementary Table 5. We analyzed the expression levels of ENTPD2 in serum exosomes from 59 colon cancer patients and 28 healthy donors (Fig. 7D and Figure S9). The serum exosomal ENTPD2 content was substantially elevated in colon cancer patients (*P* < 0.05, Fig. 7E). Moreover, an increased exosomal ENTPD2 level was correlated with an advanced TNM stage and a high tumor invasion depth (*P* < 0.05, Fig. 7F-G).

We successfully obtained tissue sections from 35 of these 59 colon cancer patients who underwent surgical resection. In these sections, we examined the correlation between the level of exosomal ENTPD2 in the serum and the number of tumor-infiltrating CD8^+^ T cells, as well as the expression level of ENTPD2 in the corresponding tumor tissues, of these 35 patients with colon cancer. Representative images of immunofluorescence staining for ENTPD2 and CD8 are presented in Fig. 7H. As expected, ENTPD2 expression was negatively correlated with tumor infiltration of CD8^+^ T cells (*r* =-0.6245, *P <* 0.001) (Fig. 7I). Interestingly, we found that the level of exosomal ENTPD2 in the serum was consistent with the ENTPD2 expression level within the tumor (*r* = 0.6736, *P <* 0.001) (Fig. 7J). Additionally, we observed a negative correlation between the exosomal ENTPD2 level in the serum of these patients and the number of CD8^+^ T cells in the corresponding tumor tissue (*r* =-0.4830, *P* = 0.0033) (Fig. 7J). Together, these observations suggest that evaluating the immune response via serum markers, such as exosomal ENTPD2, may provide a wider perspective on antitumor immune responses.

## Discussion

Due to the complexity of the TME, the efficacy of immunotherapy varies greatly among cancer patients [21]. As important immunomodulators of tumorigenesis, exosomes contribute to various characteristics of cancer, particularly in the triggering of immune escape and promotion of tumor metastasis through TME remodelling [21]. Multiple studies have demonstrated strong associations between tumor-derived exosomes carrying specific cargos and immunosuppression, which could impact the response to immune checkpoint blockade therapy [22, 23]. Here, we found for the first time that ENTPD2 is ubiquitously expressed in colon cancer cells, unlike other ATPases (CD39, ENTPD3 and ENTPD8). Our findings showed that ENTPD2 was released via exosomes from colon cancer cells to suppress CD8^+^ T-cell function by both limiting ATP-P2  X7 receptor (P2  X7R)-mediated NFATc1 nuclear transcription and promoting the activity of the adenosine-A2AR pathway (Fig. 7K). These findings suggest that ENTPD2 + exosomes could be potential targets for immunotherapy, as they contribute to destabilization of the TME.

ATP rapidly accumulates in the extracellular space because of hypoxic stress, proinflammatory signalling and cell death in the tumor core [24]. Several studies have shown the important role of ATP–adenosine metabolism in the immunopathogenesis of cancer [25–27]. Modulation of components of ATP–adenosine metabolism is a potential approach for cancer immunotherapy; options include inhibition of adenosine-generating enzymes (ATPase, AMPase), degradation of extracellular adenosine, and blockade of adenosine receptors [28–30]. Sidders [30] evaluated ATP–adenosine metabolism in different tumor types and found that colon cancer ranks second among all tumor types in terms of active adenosine signalling. The expression of ATPases, the first rate-limiting enzymes in ATP–adenosine metabolism, in colon cancer cells has rarely been reported, and further investigation is needed. Our study revealed that the expression level of CD39, a widely studied ATPase, is low in colon cancer, consistent with the findings of Bastid [6]. However, the low CD39 level within colon cancer tissues does not explain the constitutive activation of adenosine signalling [6, 30]. In addition to CD39, there are three other extracellular ATP-hydrolytic enzymes: ENTPD2, ENTPD3 and ENTPD8. We revealed that ENTPD2 expression was markedly increased in colon cancer cells. Moreover, high ENTPD2 expression was correlated with a significantly worse clinical prognosis in patients.

To date, only Chiu [31] has reported a correlation between ENTPD2 expression and M-MDSC differentiation in hepatocellular carcinoma. There are no reports on the impact of ENTPD2 on other immune cells in the TME. CD8^+^ T cells are considered the key antitumor effector cells, and we observed a negative correlation between the expression level of ENTPD2 and the number of tumor-infiltrating CD8^+^ T cells in colon cancer patients and in mouse models. Upon primary stimulation and activation, CD8^+^ T cells produce copious amounts of cytokines, which kill tumor cells. Herein, we revealed that ENTPD2 strongly suppressed the production of the cytokines IFN-γ, TNF-α, and Granzyme B by CD8^+^ T-cell subsets in MC38 tumor tissues. Furthermore, we observed significant reductions in the expression levels of cytotoxic activity-related genes in the MC38-ENTPD2 group through RNA-Seq analysis of CD8^+^ T-cell transcriptomic profiles. Together, these data suggest that high ENTPD2 expression in colon cancer cells has an inhibitory effect on the functional activity of CD8^+^ T cells in the TME.

Extracellular immunoregulatory factors can effectively modulate the function of CD8^+^ T cells in both local and distant microenvironments, thereby participating in the occurrence and development of tumors. We further discovered that ENTPD2 not only is expressed on the surface of colon cancer cell membranes but also is abundantly released into the extracellular space via exosomes. As exosomes have increasingly been accepted as crucial contributors to cancer pathogenesis, scientists have discovered certain exosomal proteins and immunoregulatory networks that are closely related to the progression of various cancers [32, 33]. One example is PD-L1-expressing melanoma cell-derived exosomes, which strongly inhibit CD8^+^ T-cell proliferation, cytokine production, and cytotoxicity [33]. Our prior investigation revealed that HMGB1 expression on tumor-derived exosomes accelerates the accumulation of TIM-1^+^ Breg cells [34]. Herein, we demonstrated that colon cancer-derived exosomes harbour ENTPD2, which can effectively inhibit CD8^+^ T-cell function. The activation of T cells involves ATP release and autocrine stimulation of the P2 receptor P2X7R to regulate Ca^2+^ influx and NFATc1 activation, leading to the secretion of interleukin-2 (IL-2) and, ultimately, T-cell proliferation [17, 18]. We proposed that exosomal ENTPD2 limits P2X7R-mediated NFATc1 activation in CD8^+^ T cells, and this assumption was confirmed by our data. In addition to increasing ATP accumulation, ENTPD2 knockdown also decreased the accumulation of adenosine. We revealed that suppression of the adenosine A2A receptor effectively abrogated the adenosine-mediated inhibitory effects on activated CD8^+^ T cells treated with ENTPD2^+^ exosomes. This confirmed the idea that ENTPD2 is expressed on exosomal surfaces and that it participates in the production of adenosine via its receptor A2AR expressed on activated CD8^+^ T cells. Therefore, even in the absence of direct exosome–CD8^+^ T-cell interactions, exosomes may still inhibit the function of CD8^+^ T cells by providing a sustained source of ENTPD2 activity. These ENTPD2^+^ exosomes inhibited P2X7R-mediated NFATc1 activation and promoted the activity of the adenosine-A2AR pathway, leading to the suppression of CD8^+^ T-cell function. These findings indicate that the absence of ENTPD2 in exosomes could reverse the immunosuppressive state.

Tissue biopsies for tumor ENTPD2 assessment are costly and invasive. In contrast, liquid biopsy provides a broader approach for monitoring ENTPD2 expression. At present, exosomes are potent candidates for assessment via liquid biopsy, mostly due to their easy purification and high stability [33]. Generally, tumor cells release considerably more exosomes than healthy cells. As a result, these exosomes are abundant in all types of body fluids, such as blood and urine [35]. Several investigations have suggested that serum exosomes and their cargos hold great promise for tumor identification and prognosis [36–40]. For example, the circulating exosomal GPC1 level is typically increased in pancreatic ductal carcinoma (PDAC) and CRC patients. Hence, it is a potent tool for detecting tumors within the digestive system [37, 38]. In NSCLC patients, circulating exosomal PD-L1 but not soluble PD-L1 is correlated with tumor progression [39]. Moreover, a high abundance of serum exosomal PD-L1 is indicative of poor prognosis in PDAC patients [40]. Herein, we quantified circulating exosomal ENTPD2 in patients with colon cancer and showed that ENTPD2 was more enriched in serum exosomes from colon cancer patients than in those from healthy controls and that a high abundance of ENTPD2 was closely associated with an advanced TNM stage and a high tumor invasion depth. The aforementioned data suggested that the relative serum exosomal ENTPD2 level was intricately linked to clinical stage and disease activity. The main advantage of using exosomal ENTPD2 as a marker is that it is stable and abundant in peripheral blood. This provides a minimally invasive method for serially assessing predictive and prognostic markers during the progression of colon cancer. We further found a significant positive correlation between the level of serum exosomal ENTPD2 and the level of ENTPD2 in paired colon cancer tissues. Additionally, exosomal ENTPD2 and tissue ENTPD2 were both negatively correlated with CD8^+^ T cell infiltration. This finding suggests that circulating ENTPD2 may reflect the tissue condition of the primary lesion to some extent. However, additional studies in large patient populations are warranted to further examine the potential of the exosomal ENTPD2 level as a biomarker for patients with colon cancer.

## Conclusion

In conclusion, we highlighted critical roles of ENTPD2 in regulating colon cancer that have not been explored before. Colon cancer cell-derived exosomes transfer ENTPD2 as an extracellular ATP scavenger, thus impairing CD8^+^ T-cell function by inhibiting P2 X7R-mediated NFATc1 activation and promoting the activity of the adenosine-A2AR pathway. Our work highlights a surprising novel role for colon cancer cell-derived exosomes, with a potential role in the propagation of metabolic signals to the surrounding TME.

### Electronic supplementary material

Below is the link to the electronic supplementary material.

Supplementary Material 1

Supplementary Material 2

Supplementary Material 3

Supplementary Material 4

## Data Availability

All data showed during this study can be downloaded.
