# Supplementary material for: Tumor derived exosomal ENTPD2 impair CD8+ T cell function in colon cancer through ATP-adenosine metabolism reprogramming
Source: Cell Commun Signal. 2024 May 16;22:274. doi: 10.1186/s12964-024-01654-2 (PMC11097558; doi:10.1186/s12964-024-01654-2)

. Images of western blots

FIG 2 A

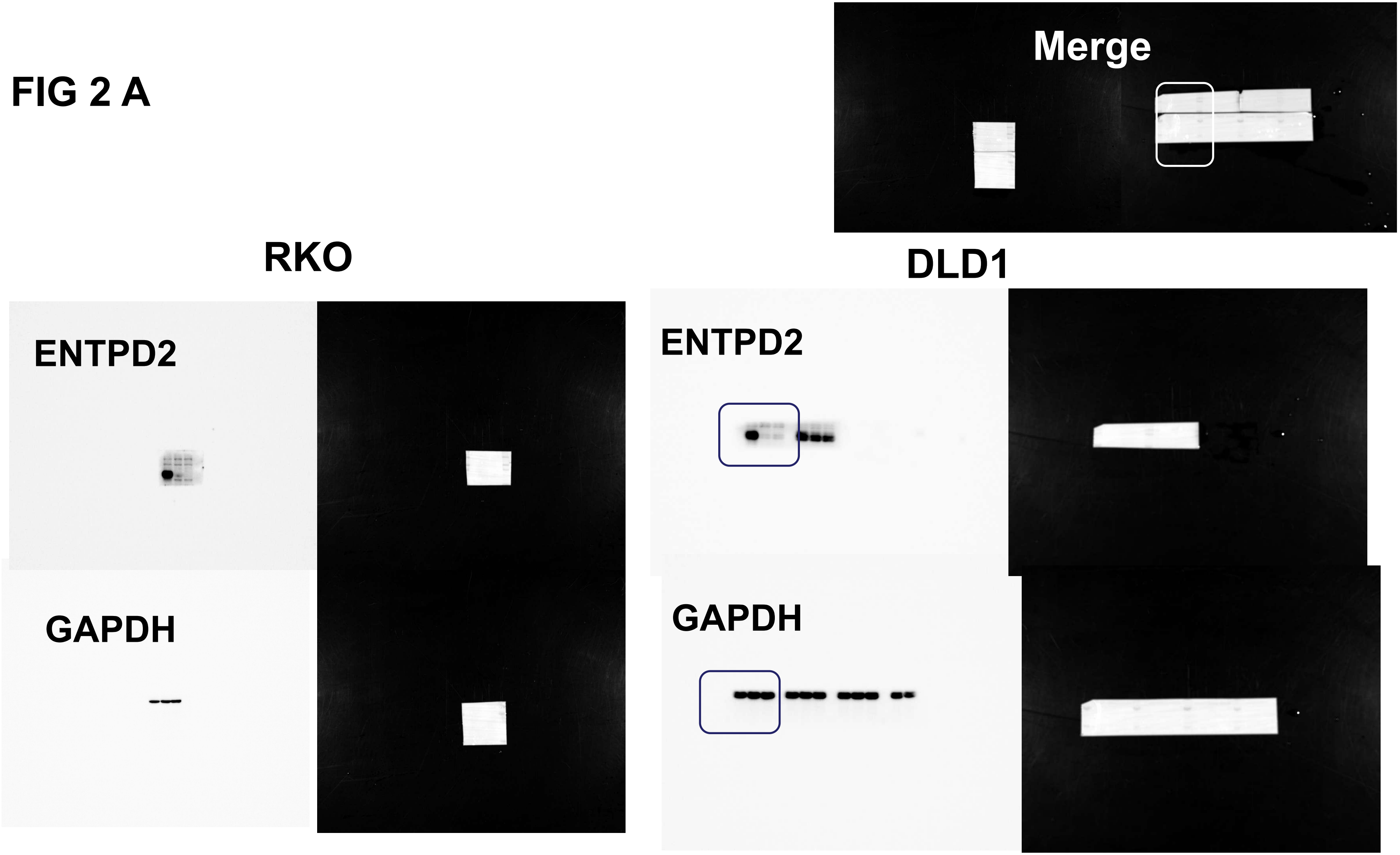

FIG 4 C

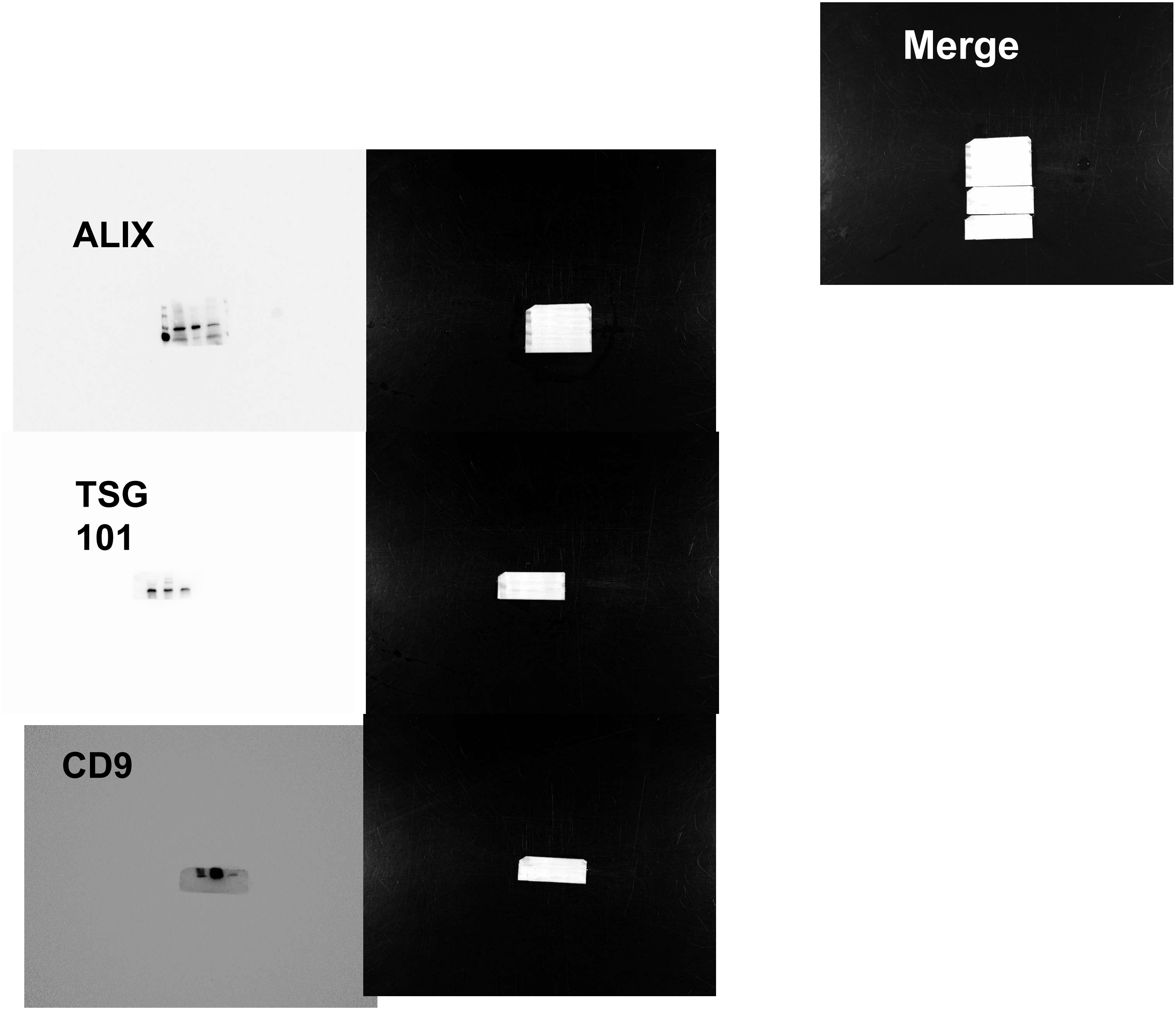

FIG 4F

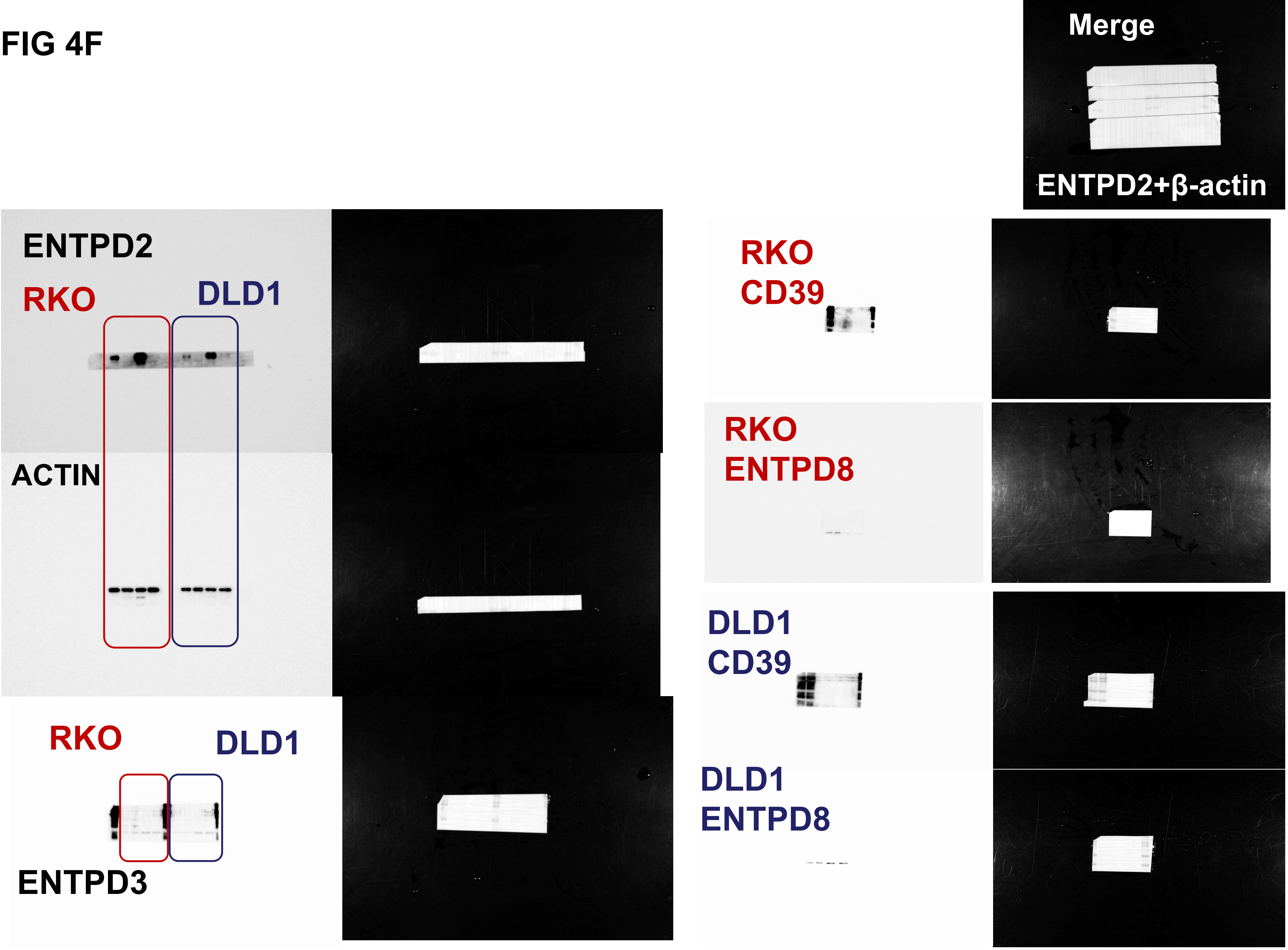
Merge
ENTPD2+β-actin
ENTPD2
RKO
DLD1
ACTIN
RKO
CD39
RKO
ENTPD8
DLD1
CD39
DLD1
ENTPD8
RKO
DLD1
ENTPD3

FIG 5 B

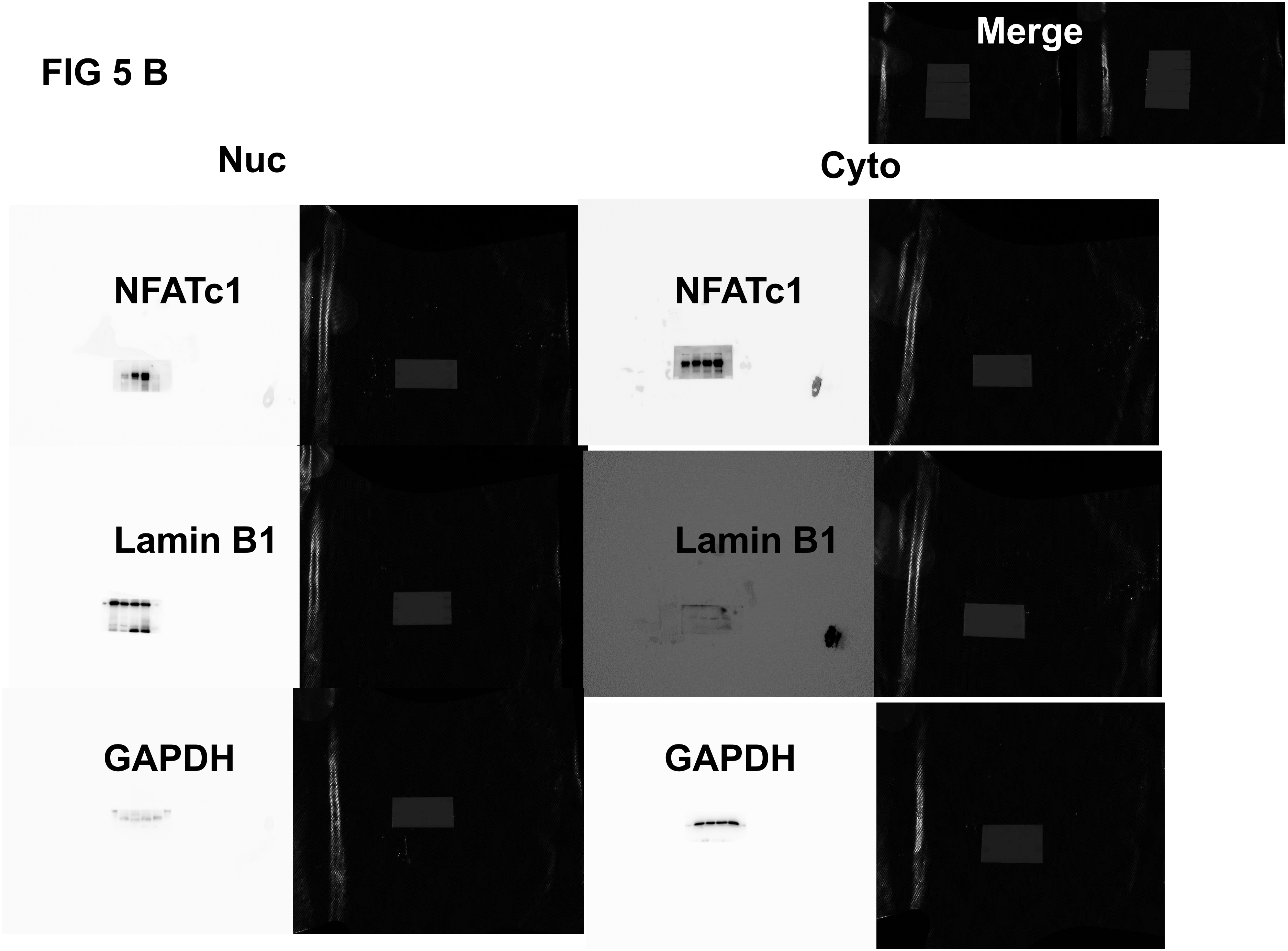

FIG 5 C

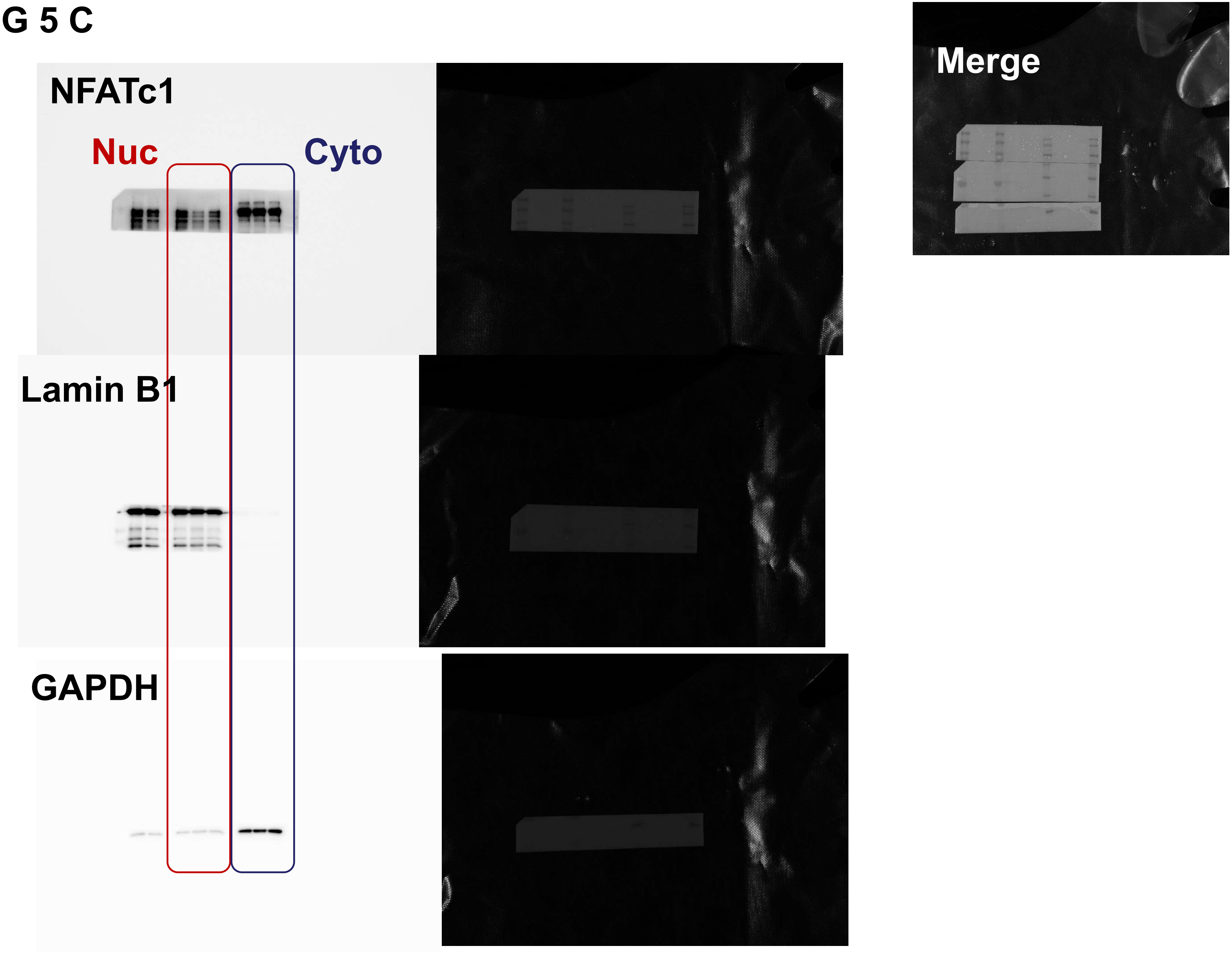

FIG 5 D

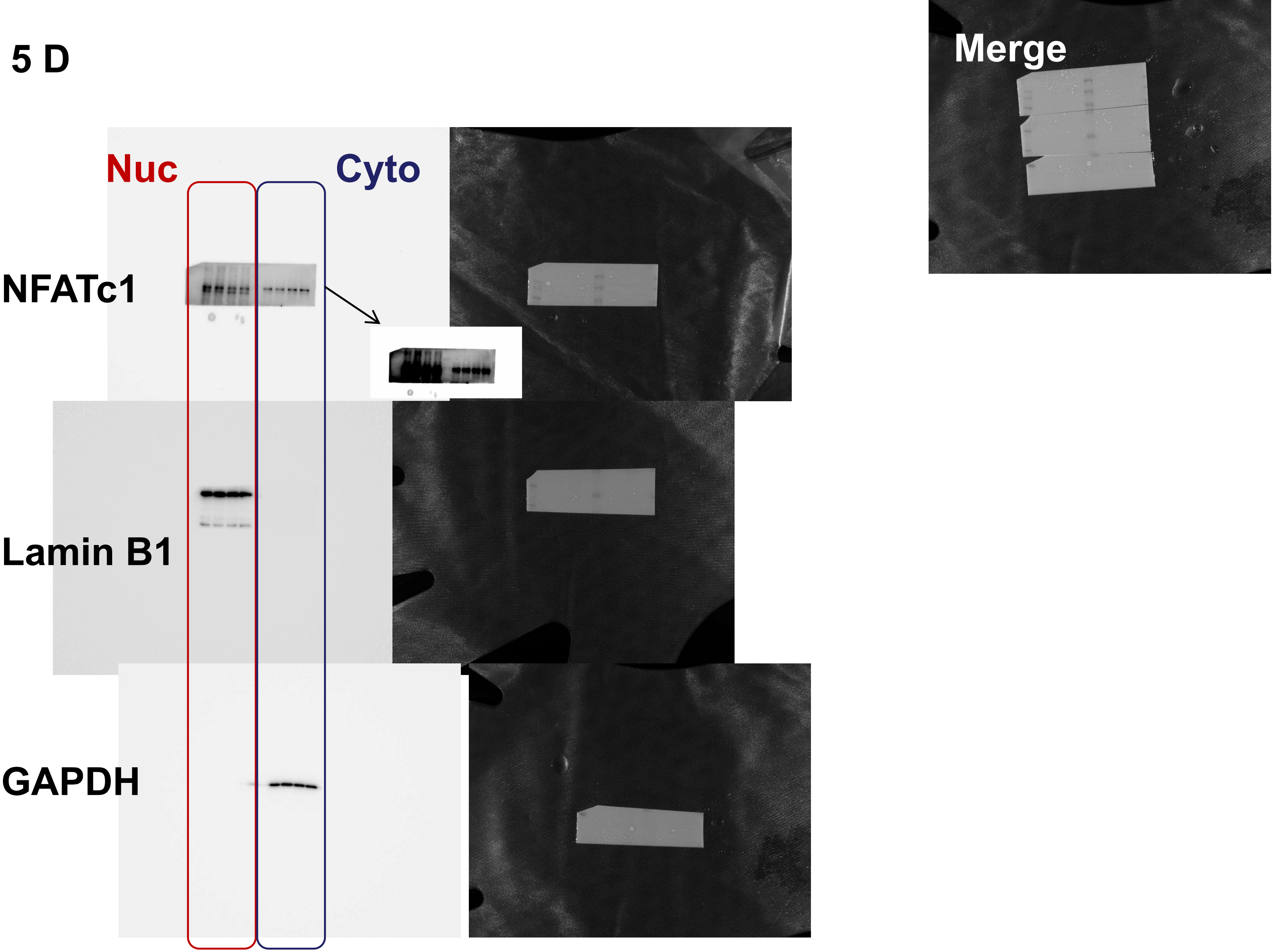

FIG 5 F

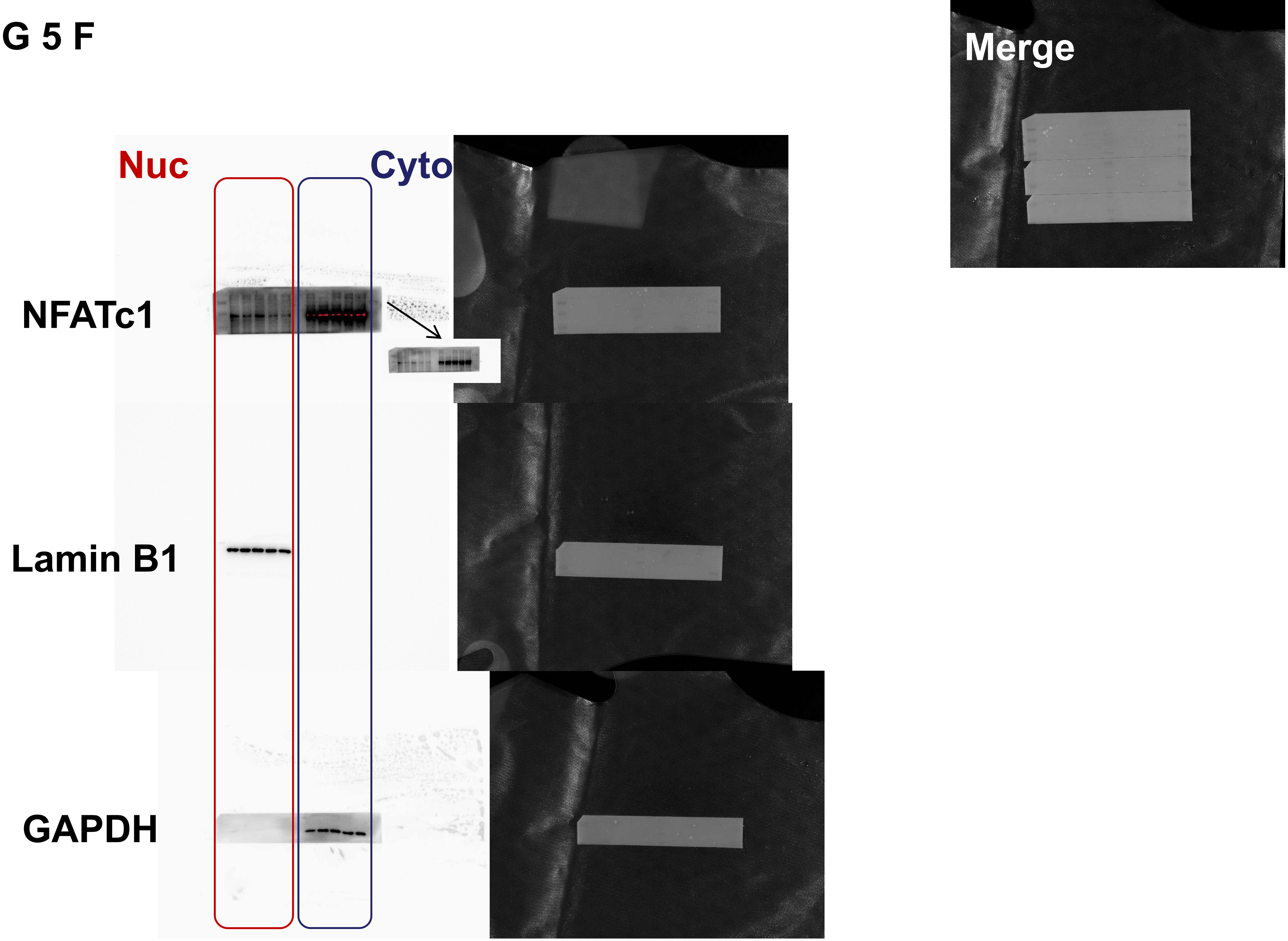

FIG 7D

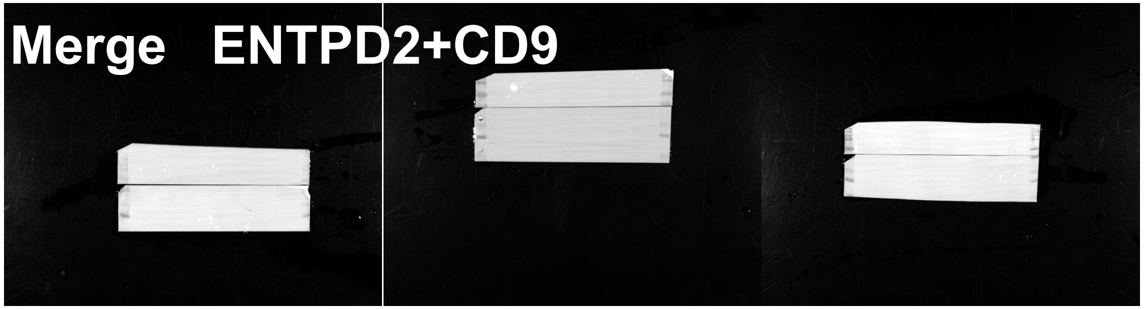

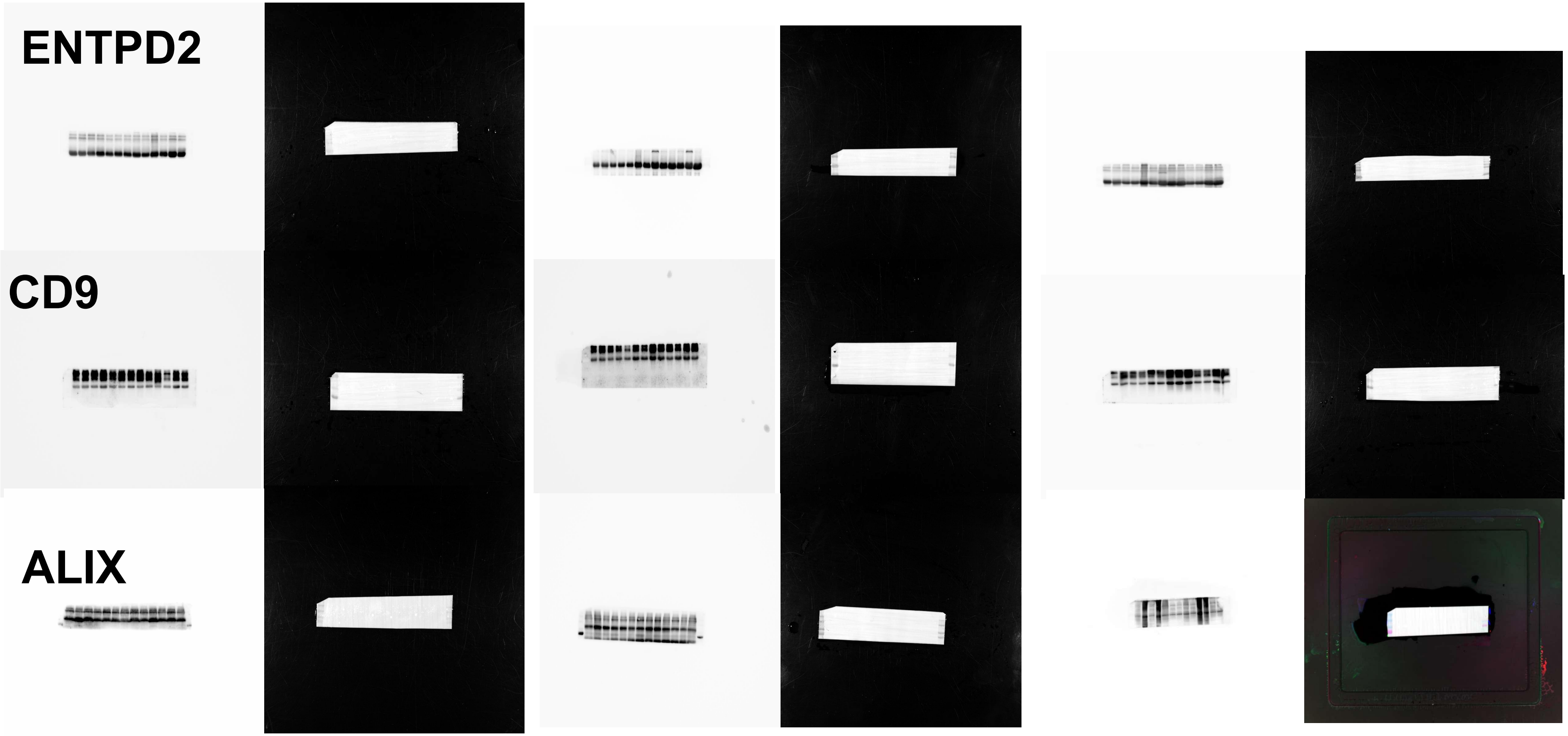

Supplemental

Fig 4A

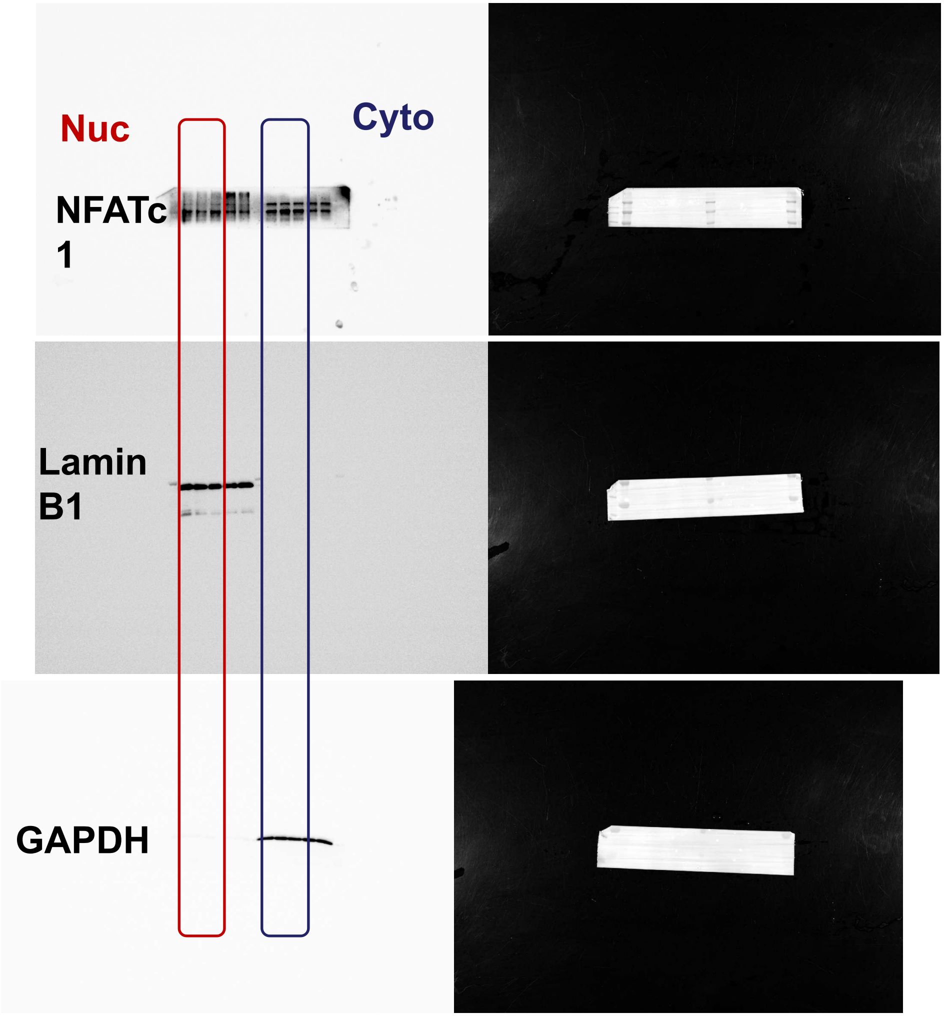

Supplemental

Fig 4B

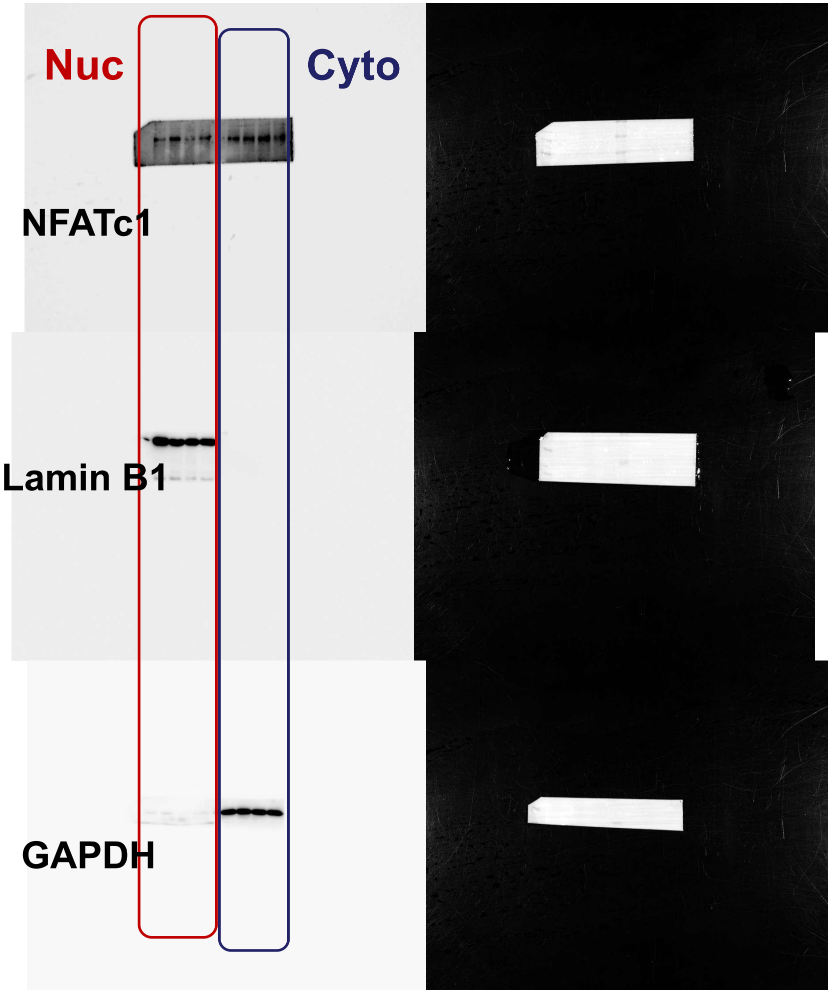

Supplemental

Fig 7

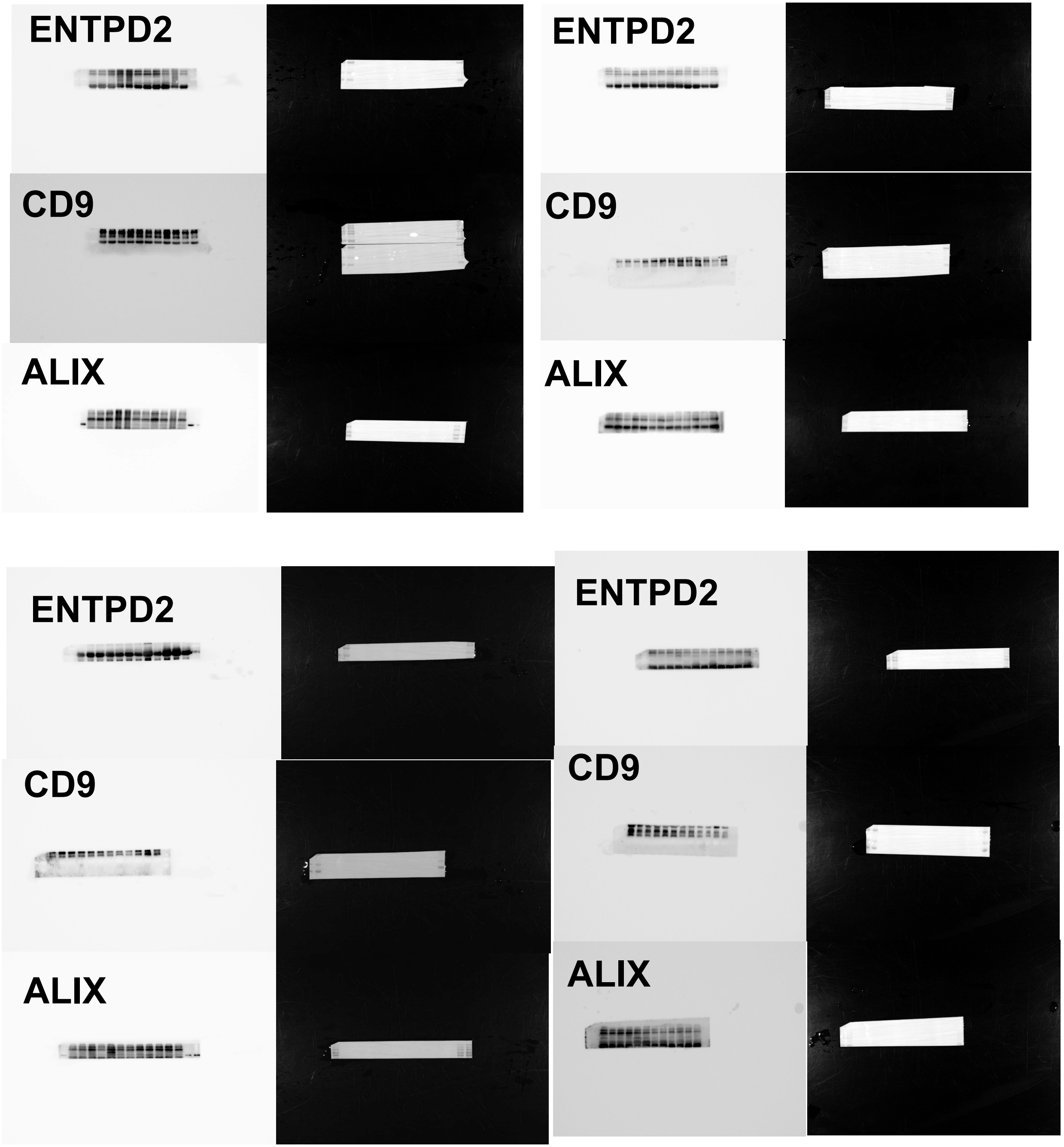

Supplement: Supplementary file 1 — Supplementary Material 1 [file 12964_2024_1654_MOESM1_ESM.pdf]
