# Supplementary material for: Tumor derived exosomal ENTPD2 impair CD8+ T cell function in colon cancer through ATP-adenosine metabolism reprogramming
Source: Cell Commun Signal. 2024 May 16;22:274. doi: 10.1186/s12964-024-01654-2 (PMC11097558; doi:10.1186/s12964-024-01654-2)
Supplement: Supplementary file 2 — Supplementary Material 2 [file 12964_2024_1654_MOESM2_ESM.doc]

**Table S1** Quantitative reverse transcription polymerase chain reaction primers.

| **Gene** | **Forward primer** | **Reverse primer** |
| --- | --- | --- |
| Human ENTPD1 | CAGGGGGATTTTGGGGCATT | TACTTCTCCTTTACTCCAGCGT |
| Human ENTPD2 | TCTTCTAAACAGCAGCATTCC | CATTTCATCCGTGTGTCTCAG |
| Human ENTPD3 | CCATTTGTGGCTTTTGCAGGA | CTGACTCCAATTCTGTGAGCA |
| Human ENTPD8 | CACAGTTGAAGGGACAGGCA | GCTGGCCTCCACATAGAACT |
| Human GAPDH | CTTCGCTCTCTGCTCCTCC | CAATACGACCAAATCCGTTG |
| Mouse ENTPD2 | GCCCTCAAGTATGGCATCGT | CGAACATCGCAAGAGCTGTG |
| Mouse GAPDH | GACATCAAGAAGGTGGTGAAGGAG | ATACCAGGAAATGAGCTTGACAAA |
|  | | |

**Table S2** The antibodies used in our experiments.

| **Species** | **Antigen** | **Product code** | | **Company** | **Dilution** | **Application** |
| --- | --- | --- | --- | --- | --- | --- |
| Human | ENTPD2 | | AF6087-SP | R&D Systems | 1:1000,1:500 | WB, IF |
| Human | CD39 | | 19229-1-AP | Proteintech | 1:1000 | WB |
| Human | ENTPD3 | | 13021-1-AP | Proteintech | 1:1000 | WB |
| Human | ENTPD8 | | 18848-1-AP | Proteintech | 1:1000 | WB |
| Human | ENTPD2 | | ab150503 | Abcam | 1:4000 | IHC |
| Human | CD8 | | ZA-0508 | ZSGB-Bio | 1:500 | IF |
| Human | GAPDH | | 5174 | Cell Signaling Technology | 1:2000 | WB |
| Human | β-actin | | 66009-1-Ig | Proteintech | 1:2000 | WB |
| Human | NFAT2 | | 8032 | Cell Signaling Technology | 1:1000 | WB |
| Human | Lamin B1 | | 12987-1-AP | Proteintech | 1:5000 | WB |
| Human | CD9 | | ab92726 | Abcam | 1:500 | WB |
| Human | TSG101 | | ab125011 | Abcam | 1:500 | WB |
| Human | ALIX | | ab275377 | Abcam | 1:500 | WB |
| Mouse | ENTPD2 | | 13656-1-AP | Proteintech | 1:1000 | WB, IHC |
| Mouse | CD8 | | ab217344 | Abcam | 1:2000 | IHC |
| Mouse | CD3 | | ab135372 | Abcam | 1:2000 | IHC |
| Mouse | CD4 | | 67786-1-Ig | Proteintech | 1:2000 | IHC |
| Mouse | CD68 | | 30929-1-AP | Proteintech | 1:1000 | IHC |
| Mouse | MPO | | GB150006 | Servicebio | 1:1000 | IHC |

**Table S3** Flow cytometry panels for analysis of immune cells.

| **Species** | **Antigen** | **Fluorochrome** | **Product code** | **Company** |
| --- | --- | --- | --- | --- |
| Mouse | CD8 | BV605 | Cat# 100743 | Biolegend |
| Mouse | Granzyme B | APC | Cat# 372204 | Biolegend |
| Mouse | TNF-α | BV421 | Cat# 506327 | Biolegend |
| Mouse | IFN-γ | FITC | Cat# 505806 | Biolegend |
| Mouse | PD-1 | PE | Cat# 135205 | Biolegend |
| Mouse | CD44 | PerCP/Cy5.5 | Cat# 103031 | Biolegend |
| Human | CD3 | PE | Cat# 300308 | Biolegend |
| Human | CD8 | FITC, APC | Cat# 344704, 344722 | Biolegend |
| Human | Granzyme B | FITC | Cat# 372206 | Biolegend |
| Human | TNF-α | PE-Cy7 | Cat# 502930 | Biolegend |
| Human | IFN-γ | APC-Cy7 | Cat# 502530 | Biolegend |
| Human | CD63 | FITC | Cat# 561924 | BD Biosciences |
| Human | CD81 | FITC | Cat# 551108 | BD Biosciences |

**Table S4**  Correlations of ENTPD2 protein levels with clinicopathological variables in colon cancer.

| Factors | Number of cases (*n*) | ENTPD2 | | *P* |
| --- | --- | --- | --- | --- |
|  |  | Low | High |  |
| Age (years) |  | | | 0.936 |
| ＜ 60 | 30 | 15(50.0%) | 15(50.0%) |  |
| ≥ 60 | 55 | 28(50.9%) | 27(49.1%) |  |
| Gender |  | | | 0.450 |
| Female | 44 | 24(54.5%) | 20(45.5%) |  |
| Male | 41 | 19(46.3%) | 22(53.7%) |  |
| pT |  | | | 0.050 |
| T1＋T2 | 9 | 8(88.9%) | 1(11.1%) |  |
| T3 | 29 | 13(44.8%) | 16(55.2%) |  |
| T4 | 47 | 22(46.8%) | 25(53.2%) |  |
| pN |  | | | 0.035 |
| N0 | 54 | 32(59.3%) | 22(40.7%) |  |
| N1+N2 | 31 | 11(35.5%) | 20(64.5%) |  |
| pM |  | | | 0.981 |
| M0 | 81 | 41(50.6%) | 40(49.4%) |  |
| M1 | 4 | 2(50.0%) | 2(50.0%) |  |
| TNM stage |  | | | 0.037 |
| Ⅰ＋Ⅱ | 52 | 31(59.6%) | 21(40.4%) |  |
| Ⅲ＋Ⅳ | 33 | 12(36.4%) | 21(63.6%) |  |

pT represents depth of tumor invasion; pN represents lymph node metastases; pM represents distant metastasis. ^*^*P* < 0.05.

**Table S5**  Clinicopathologic parameters of involved colon cancer patients.

| Characteristics | Number of cases (*n*) |
| --- | --- |
|
| Age (years) |  |
| ＜ 60 | 31 |
| ≥ 60 | 28 |
| Gender |  |
| Female | 23 |
| Male | 36 |
| TNM stage |  |
| I＋Ⅱ | 34 |
| Ⅲ＋Ⅳ | 25 |
| pT |  |
| T1+T2＋T3 | 44 |
| T4 | 15 |
| pN |  |
| N0 | 24 |
| N1+N2 | 35 |
| pM |  |
| M0 | 44 |
| M1 | 15 |

pT represents depth of tumor invasion; pN represents lymph node metastases; pM represents distant metastasis.
