# Supplementary material for: Tumor derived exosomal ENTPD2 impair CD8+ T cell function in colon cancer through ATP-adenosine metabolism reprogramming
Source: Cell Commun Signal. 2024 May 16;22:274. doi: 10.1186/s12964-024-01654-2 (PMC11097558; doi:10.1186/s12964-024-01654-2)
Supplement: Supplementary file 3 — Supplementary Material 3 [file 12964_2024_1654_MOESM3_ESM.doc]

**Supplemental Fig**

**
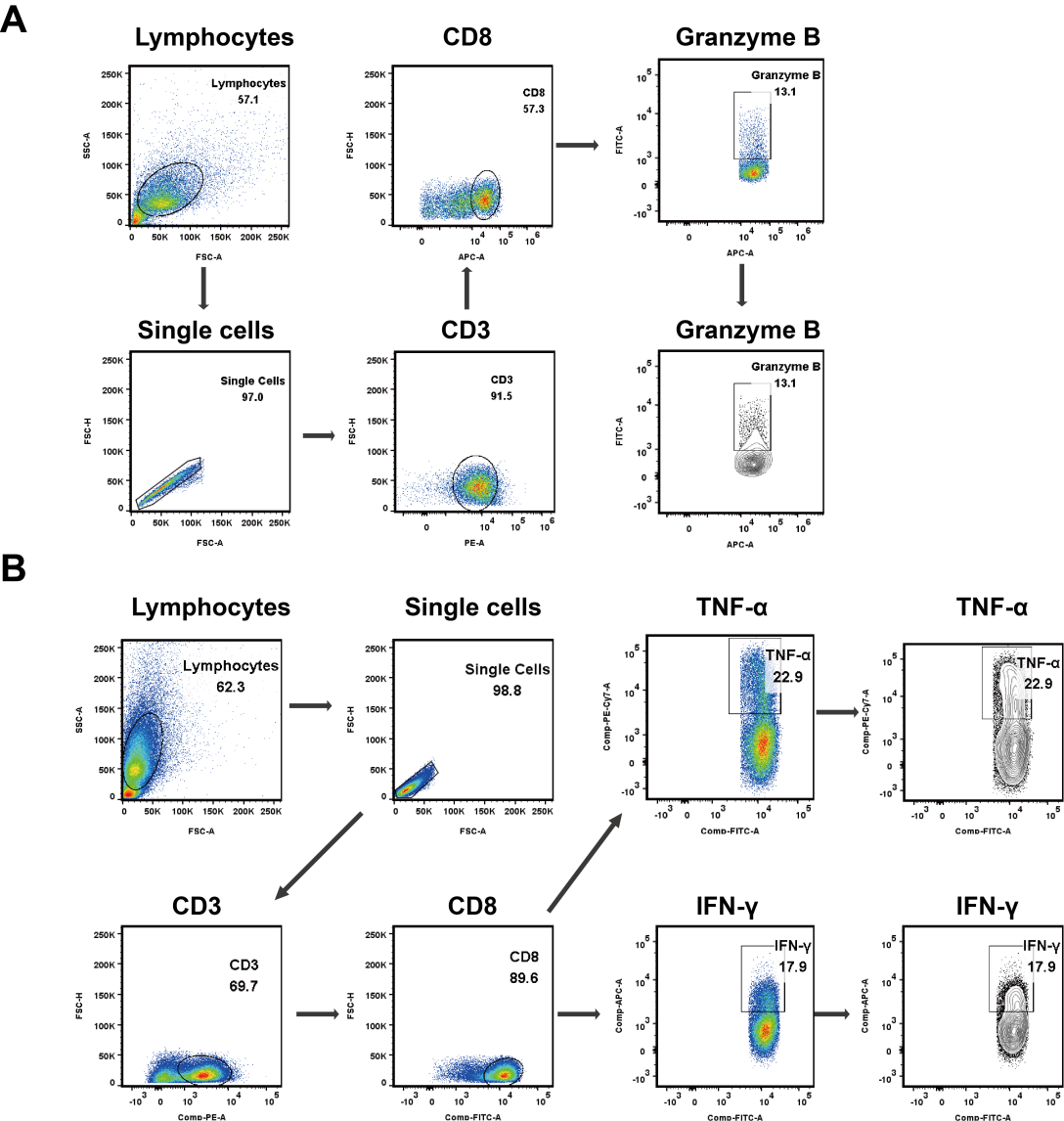
**

**Figure S1 Gating strategy for the analysis of CD8^+^ T cells.**

**A** The gating strategy for identifying granzyme B-producing CD8^+^ T cells isolated from PBMC. **B** The gating strategy for identifying cytokine-producing CD8^+^ T cells isolated from PBMC.

**
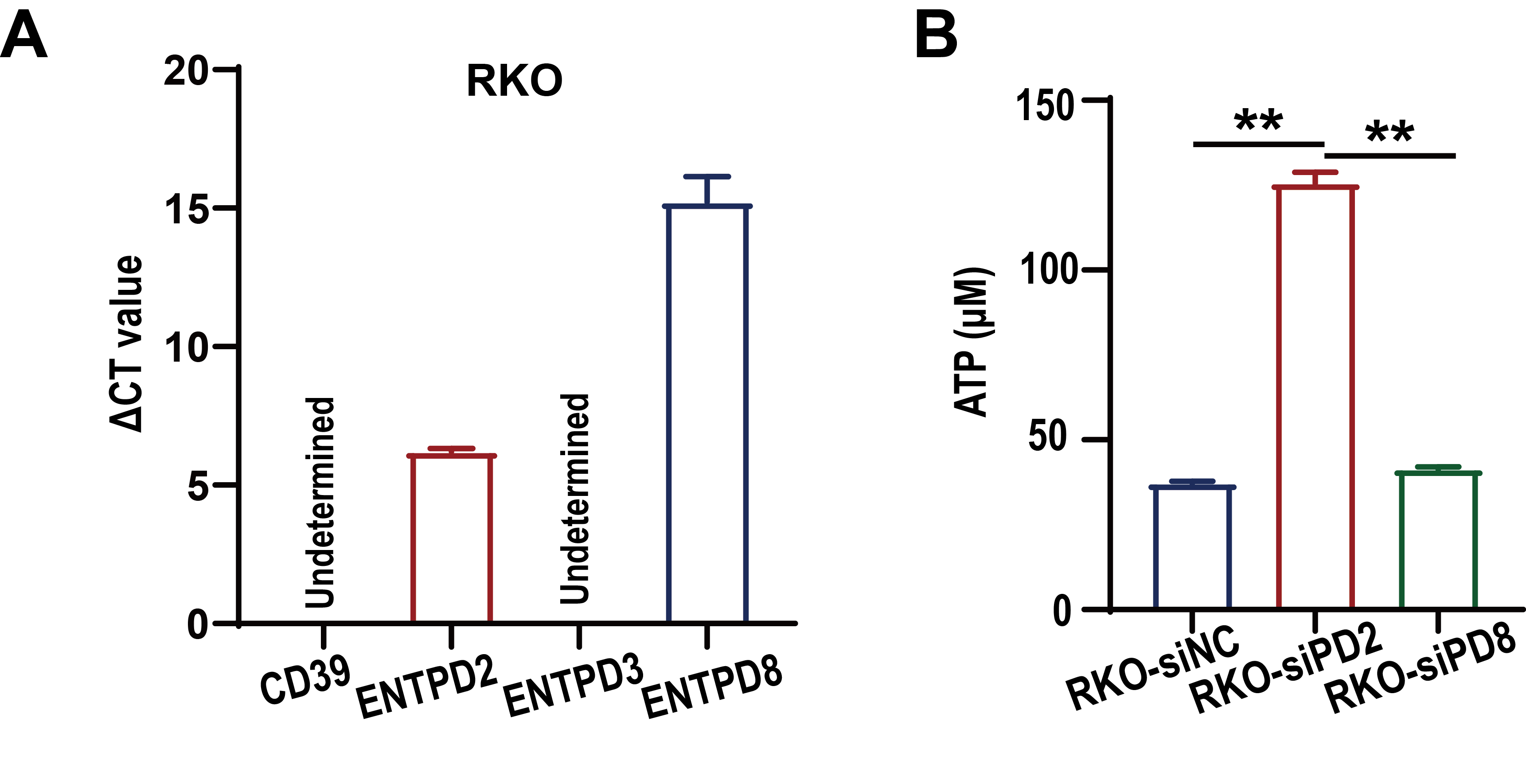
**

**Figure S2 ENTPD2 is highly expressed in colon cancer cell RKO.**

**A** The mRNA expression data displayed as △Ct values for extracellular ENTPDase members (CD39, ENTPD2, ENTPD3, and ENTPD8) in RKO cell. **B** ATP hydrolytic activity was analyzed in the culture medium from RKO-siNC, RKO-siPD2 and RKO-siPD8 cells followed by 150 μM ATP treatment for 2 h. One-way ANOVA test was performed for statistical analysis; ^**^*P* < 0.01.

**
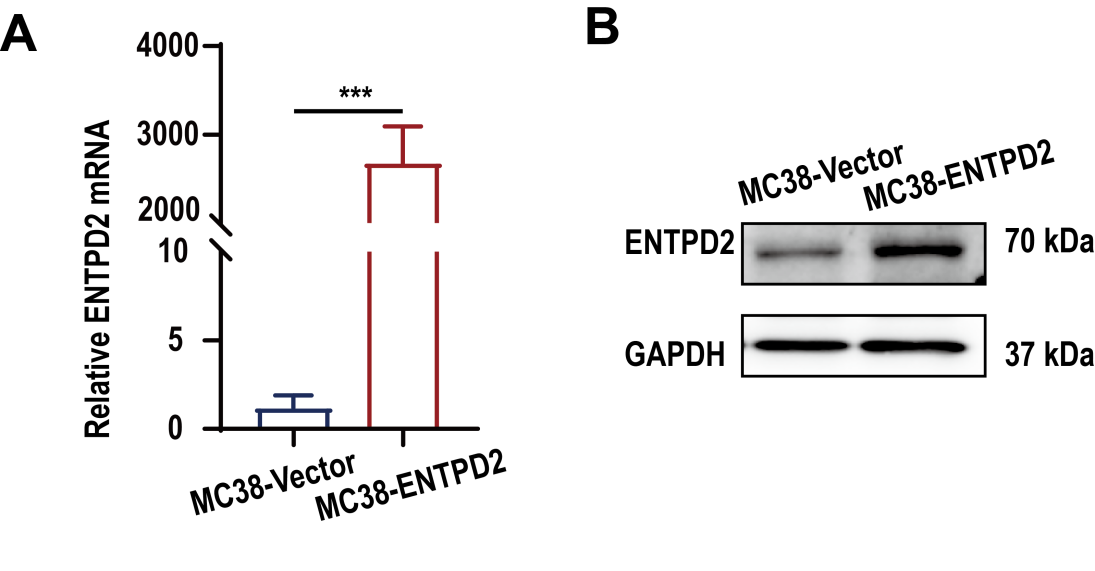
**

**Figure S3 Representative qRT-PCR and western blotting results of ENTPD2 in MC38 cells from the stable ENTPD2 overexpression (MC38-ENTPD2) group and the vector control (MC38-Vector) group.**

**
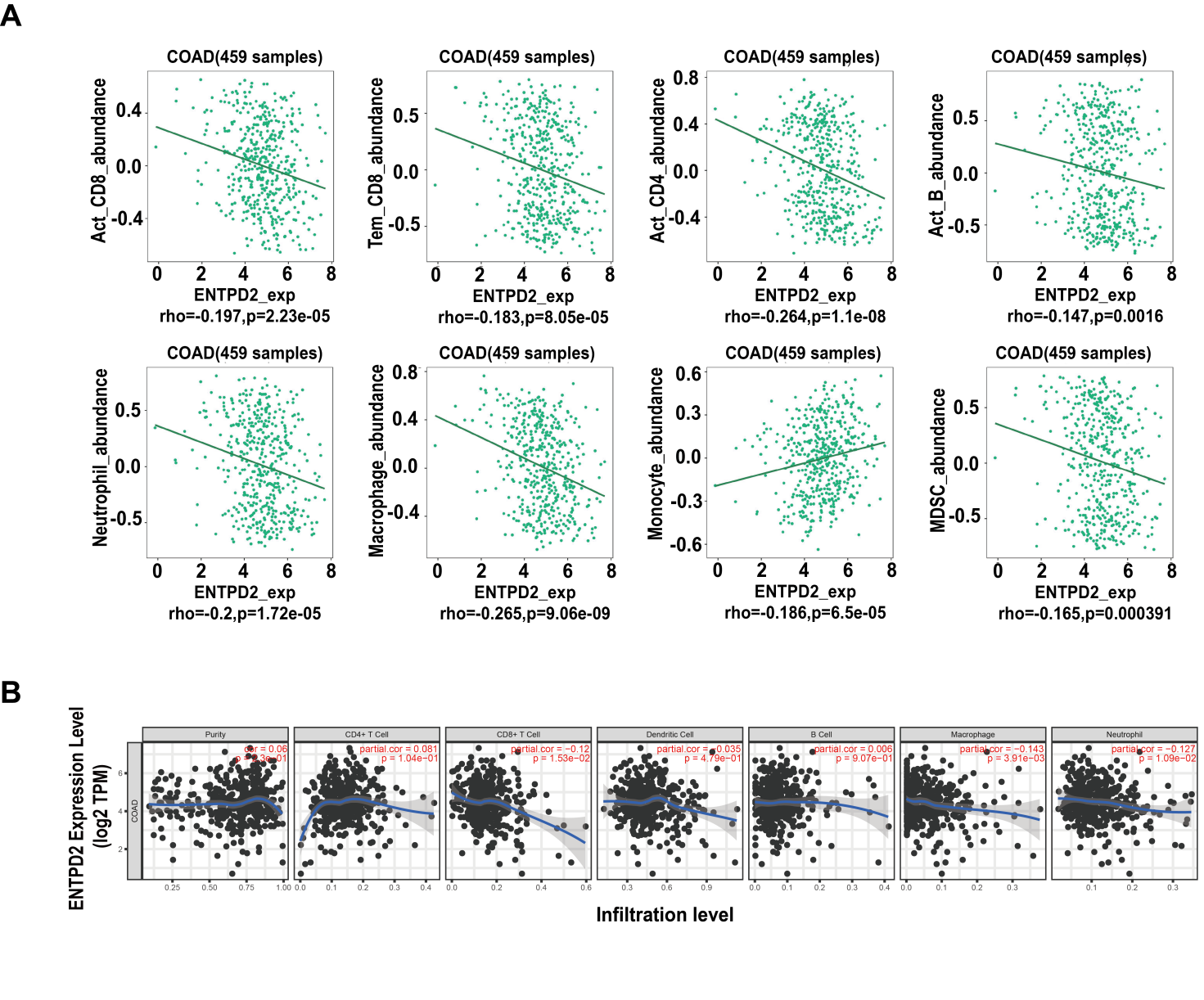
**

**Figure S4 Correlation between ENTPD2 expression level and the degree of various immune cells infiltration in colon cancer.**

**A** Correlation between ENTPD2 expression and immune infiltration levels in colon adenocarcinoma from the TISIDB website. **B** Correlation between ENTPD2 expression and immune infiltration levels in colon adenocarcinoma from the TIMER database.

**
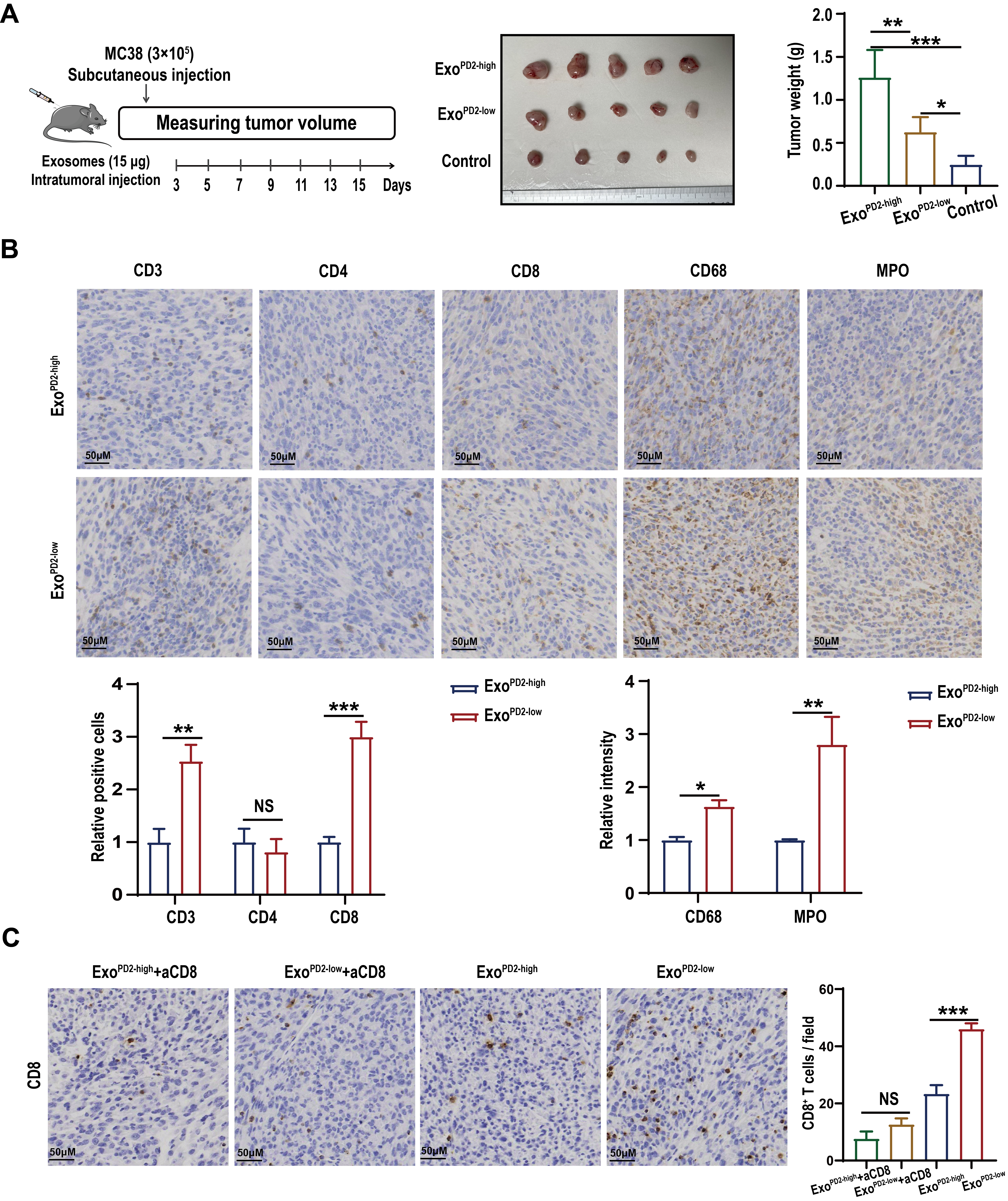
**

**Figure S5 Exosomal ENTPD2 derived from colon cancer cells promote tumor growth.**

**A** Representative images and weights of MC38 tumors from C57BL/6 mice after treatment with PBS, Exo^PD2-high^ or Exo^PD2-LOW^. **B** Immunohistochemical staining for CD3, CD4, CD8, CD68 and MPO in sections of mouse tumor tissues from the Exo^PD2-high^ group and Exo^PD2-LOW^ group. **C** Immunohistochemical staining for CD8 in sections of mouse tumor tissues from C57BL/6 mice treated with an anti-CD8a or isotype control antibody followed by treatment with exosomes (Exo^PD2-high^ or Exo^PD2-LOW^). **A,C** One-way ANOVA was used for statistical analysis. **B** Student’s *t* test was used for statistical analysis; ^*^*P* < 0.05, ^**^*P* < 0.01, ^***^*P* < 0.001, NS: not significant.

**
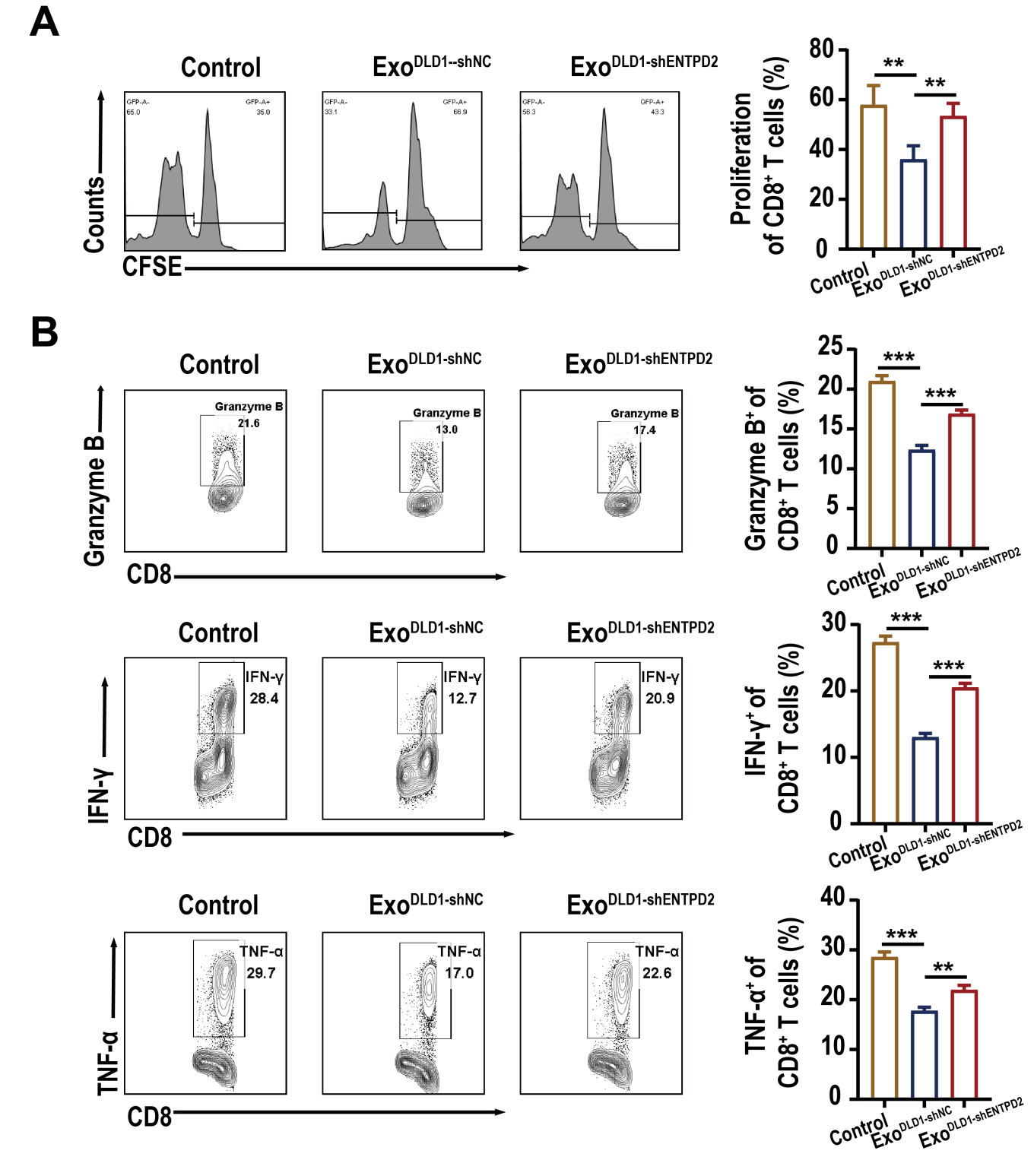
**

**Figure S6 ENTPD2 in exosomes suppresses CD8^+^ T cell proliferation and cytotoxicity.**

**A** Representative proliferation histograms of stimulated CD8^+^ T cells incubated with exosomes from DLD1 cell (Exo^DLD1- shNC^ or Exo^DLD1-shENTPD2^). **B** Representative contour plots of stimulated CD8^+^ T cells examined for the expression of Granzyme B, IFN-γ and TNF-α after incubation with Exo^DLD1-shNC^ or Exo^DLD1-shENTPD2^. One-way ANOVA test was performed for statistical analysis; ^**^*P* < 0.01, ^***^*P* < 0.001.

**
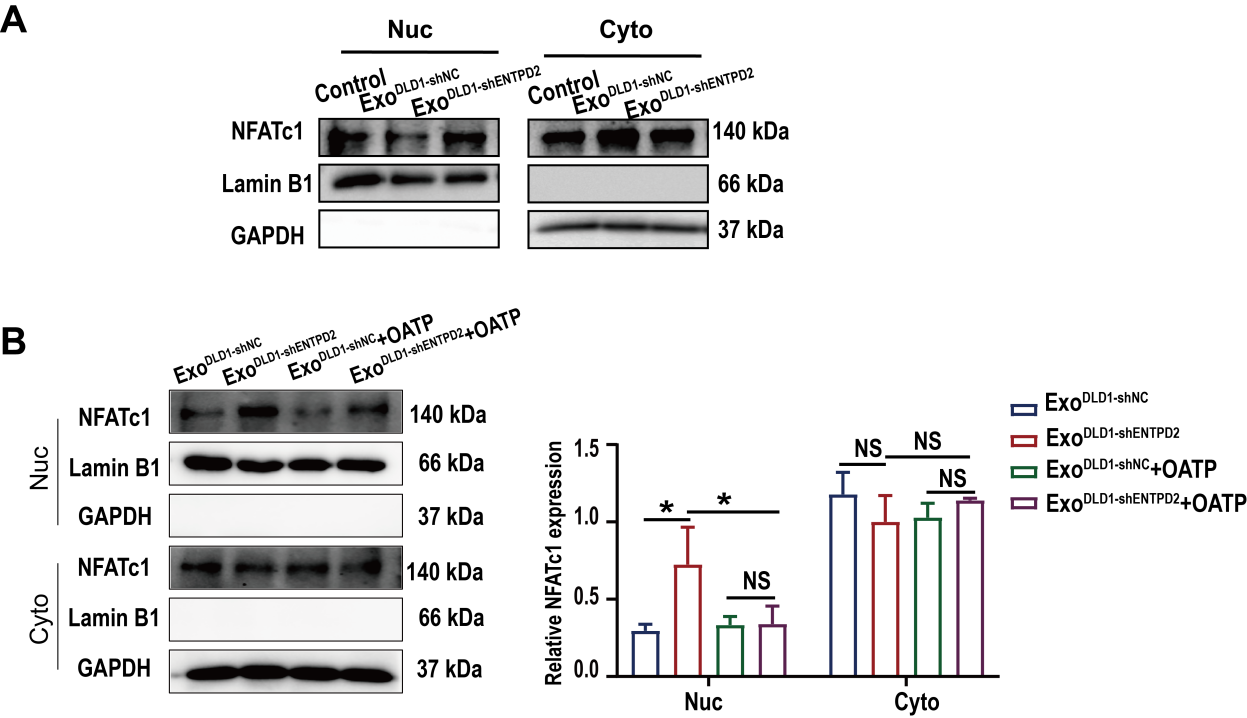
**

**Figure S7 Exosomal ENTPD2 affects NFATc1 activation of CD8^+^ T cells through the eATP-P2X7R pathway.**

**A** Western blotting for the activation of NFATc1 in stimulated CD8+ T cells incubated with exosomes from DLD1 cell (Exo^DLD1-shNC^ or Exo^DLD1-shENTPD2^). **B** Western blotting to assess NFATc1 activation in stimulated CD8^+^ T cells incubated with exosomes from colon cancer cells in the presence or absence of P2X7R antagonists. Human CD8^+^ T cells were prestimulated with anti-CD3/CD28 mAb, then incubated with 30 μg/mL Exo^DLD1-shNC^ or Exo^DLD1-shENTPD2^ in the presence or absence of the P2X7R antagonist OATP (300 μM) .

**
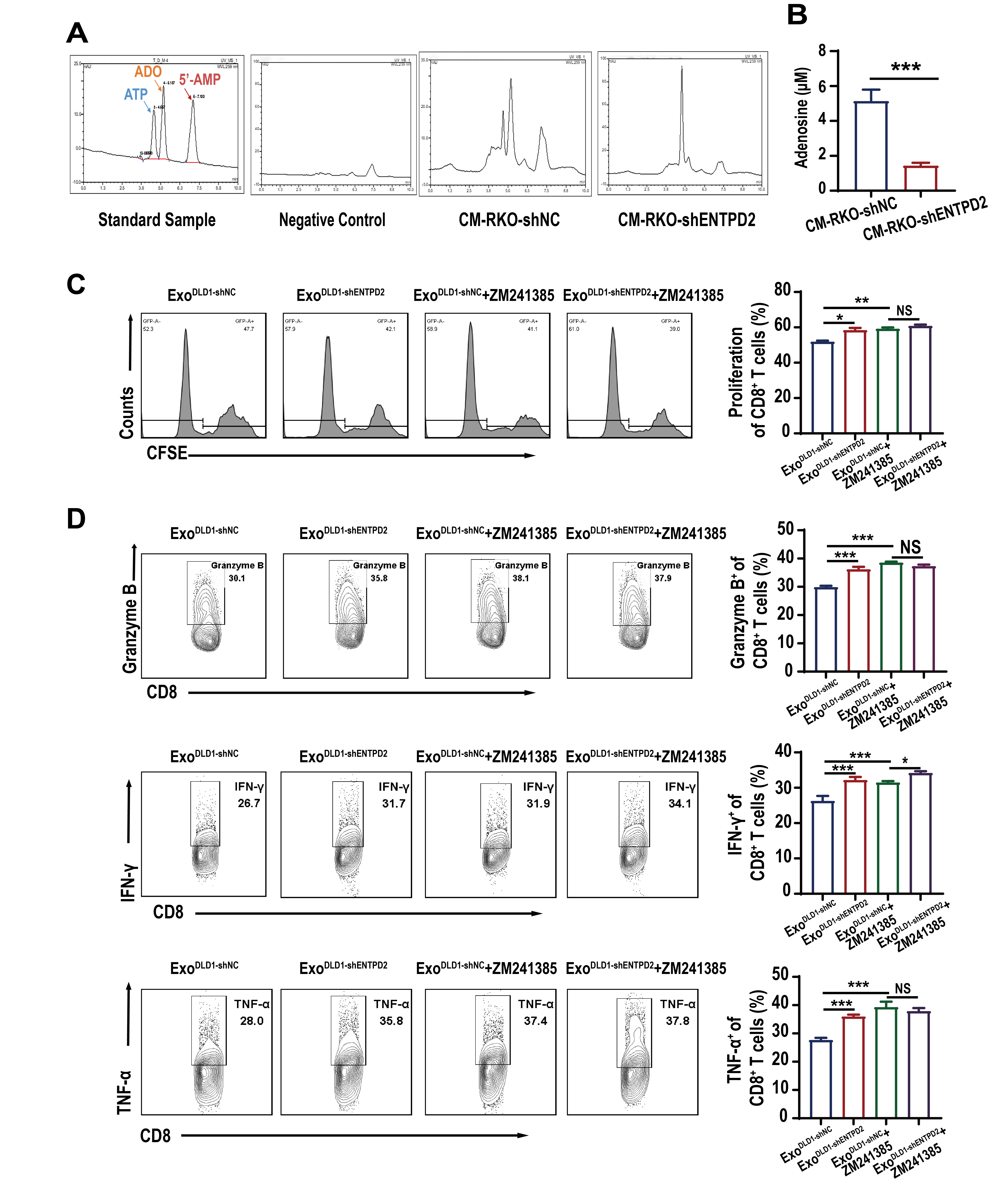
**

**Figure S8 Exosomal ENTPD2 is involved in the suppression of CD8^+^ T cell proliferation and cytotoxicity via adenosine-A2AR pathway.**

**A** The concentrations of ATP, 5’-AMP and adenosine were measured by HPLC in standard sample (standard solution), the negative control (CM from RKO cells cultured in the absence of ATP), CM-RKO-shNC (CM from RKO-shNC cells cultured in the presence of ATP) and CM-RKO-shENTPD2 (CM from RKO-shENTPD2 cells cultured in the presence of ATP). **B** The concentration of adenosine was analysed in CM-RKO-shNC and CM-RKO-shENTPD2 (CM from RKO-shNC or RKO-shENTPD2 cells cultured in the presence of ATP). **C** Representative proliferation histograms of anti-CD3/CD28 mAb stimulated CD8^+^ T cells cocultured in the presence of 30 μg/mL Exo^DLD1-shNC^ or Exo^DLD1-shENTPD2^ with or without ZM241385. **D** Granzyme B, IFN-γ and TNF-α expression evaluated with flow cytometry in anti-CD3/CD28 mAb stimulated CD8^+^ T cells after incubation with 30 μg/mL Exo^DLD1-shNC^ or Exo^DLD1-shENTPD2^ in the presence or absence of ZM241385. **B** Student’s *t* test was used for statistical analysis; **C, D** One-way ANOVA test was performed for statistical analysis; ^*^*P* < 0.05, ^**^*P* < 0.01, NS: not significant.


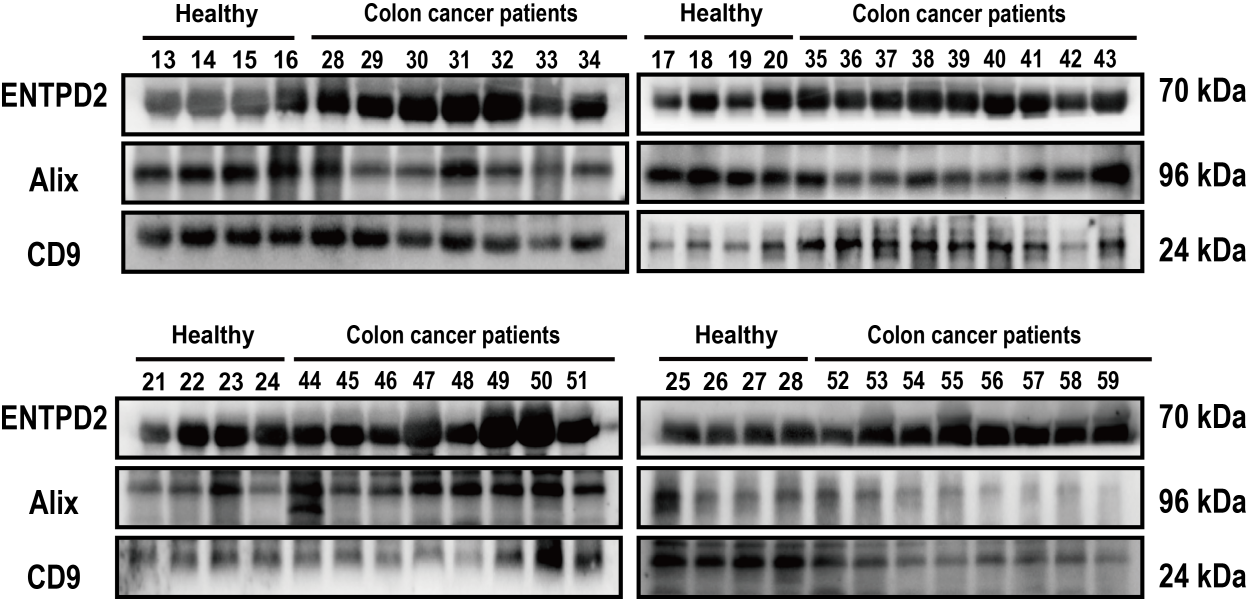


**Figure S9 The levels of ENTPD2 in exosomes isolated from the serum of colon cancer patients and healthy individuals.**

Representative western blotting for ENTPD2 and typical exosomal markers (Alix, CD9) in exosomes isolated from the serum of colon cancer patients. Exosomes isolated from healthy individuals served as normal controls.
