# Supplementary material for: Tumor derived exosomal ENTPD2 impair CD8+ T cell function in colon cancer through ATP-adenosine metabolism reprogramming
Source: Cell Commun Signal. 2024 May 16;22:274. doi: 10.1186/s12964-024-01654-2 (PMC11097558; doi:10.1186/s12964-024-01654-2)
Supplement: Supplementary file 4 — Supplementary Material 4 [file 12964_2024_1654_MOESM4_ESM.pdf]

SPRINGER NATURE
Author Services

Editing Certificate

This document certifies that the manuscript

Tumor derived exosomal ENTPD2 impair CD8+ T cell function in colon cancer through ATP-adenosine metabolism reprogramming

prepared by the authors

Mengchen Shi, Linsen Ye, Lu Zhao, Lingyuan He, Junxiong Chen, Jingdan Zhang, Yixi Su, Haiyan Dong, Jiaqi Liu, Liumei Liang, Wenwen Zheng, Yanhong Xiao, Huanliang Liu, Xiangling Yang, Zihuan Yang.

was edited for proper English language, grammar, punctuation, spelling, and overall style by one or more of the highly qualified native English speaking editors at SNAS.

This certificate was issued on **April 3, 2024** and may be verified on the SNAS website using the verification code **DB68-2A62-A757-C56A-BC5F**.

Neither the research content nor the authors' intentions were altered in any way during the editing process. Documents receiving this certification should be English-ready for publication; however, the author has the ability to accept or reject our suggestions and changes. To verify the final SNAS edited version, please visit our verification page at secure.authorservices.springernature.com/certificate/verify. If you have any questions or concerns about this edited document, please contact SNAS at support@as.springernature.com.

SNAS provides a range of editing, translation, and manuscript services for researchers and publishers around the world. For more information about our company, services, and partner discounts, please visit authorservices.springernature.com.
